# The role of low-volatility organic compounds in initial particle growth in the atmosphere

**DOI:** 10.1038/nature18271

**Published:** 2016-05-25

**Authors:** Jasmin Tröstl, Wayne K. Chuang, Hamish Gordon, Martin Heinritzi, Chao Yan, Ugo Molteni, Lars Ahlm, Carla Frege, Federico Bianchi, Robert Wagner, Mario Simon, Katrianne Lehtipalo, Christina Williamson, Jill S. Craven, Jonathan Duplissy, Alexey Adamov, Joao Almeida, Anne-Kathrin Bernhammer, Martin Breitenlechner, Sophia Brilke, Antònio Dias, Sebastian Ehrhart, Richard C. Flagan, Alessandro Franchin, Claudia Fuchs, Roberto Guida, Martin Gysel, Armin Hansel, Christopher R. Hoyle, Tuija Jokinen, Heikki Junninen, Juha Kangasluoma, Helmi Keskinen, Jaeseok Kim, Manuel Krapf, Andreas Kürten, Ari Laaksonen, Michael Lawler, Markus Leiminger, Serge Mathot, Ottmar Möhler, Tuomo Nieminen, Antti Onnela, Tuukka Petäjä, Felix M. Piel, Pasi Miettinen, Matti P. Rissanen, Linda Rondo, Nina Sarnela, Siegfried Schobesberger, Kamalika Sengupta, Mikko Sipilä, James N. Smith, Gerhard Steiner, Antònio Tomè, Annele Virtanen, Andrea C. Wagner, Ernest Weingartner, Daniela Wimmer, Paul M. Winkler, Penglin Ye, Kenneth S. Carslaw, Joachim Curtius, Josef Dommen, Jasper Kirkby, Markku Kulmala, Ilona Riipinen, Douglas R. Worsnop, Neil M. Donahue, Urs Baltensperger

**Affiliations:** 1 Paul Scherrer Institute, Laboratory of Atmospheric Chemistry, Villigen, CH-5232 Switzerland; 2 Carnegie Mellon University, Center for Atmospheric Particle Studies, Pittsburgh, 15213 Pennsylvania USA; 3 CERN, Geneva, CH-1211 Switzerland; 4 Goethe University Frankfurt, Institute for Atmospheric and Environmental Sciences, Frankfurt am Main, 60438 Germany; 5 Department of Physics, University of Helsinki, PO Box 64, Helsinki, FI-00014 Finland; 6 Department of Applied Environmental Science, University of Stockholm, Stockholm, SE-10961 Sweden; 7 Institute for Atmospheric and Climate Science, ETH Zürich, Zürich, 8092 Switzerland; 8 Chemical Sciences Division, Earth System Research Laboratory, NOAA, Boulder, Colorado USA; 9 Division of Chemistry and Chemical Engineering, California Institute of Technology, Pasadena, 91125 California USA; 10 Helsinki Institute of Physics, University of Helsinki, PO Box 64, Helsinki, FI-00014 Finland; 11 Institute for Ion and Applied Physics, University of Innsbruck, Innsbruck, 6020 Austria; 12 Ionicon Analytik GmbH, Innsbruck, 6020 Austria; 13 WSL Institute for Snow and Avalanche Research SLF, Davos, 7260 Switzerland; 14 University of Eastern Finland, Kuopio, 70211 Finland; 15 Finnish Meteorological Institute, Helsinki, 00101 Finland; 16 National Center for Atmospheric Research, Atmospheric Chemistry Observations and Modeling Laboratory, Boulder, 80301 Colorado USA; 17 Institute of Meteorology and Climate Research, Karlsruhe Institute of Technology, Karlsruhe, Germany; 18 School of Earth and Environment, University of Leeds, Leeds, LS2 9JT UK; 19 Department of Chemistry, University of California, Irvine, 92697 California USA; 20 Faculty of Physics, University of Vienna, Vienna, 1090 Austria; 21 SIM, University of Lisbon and University of Beira Interior, Lisbon, 1849-016 Portugal; 22 Aerodyne Research, Inc., Billerica, 01821 Massachusetts USA; 23 Present Address: † Present addresses: Cooperative Institute for Research in Environmental Sciences, University of Colorado Boulder, Boulder, Colorado, USA and Chemical Sciences Division NOAA Earth System Research Laboratory, Boulder, Colorado, USA (C.W.); SMEAR II, Hyytiälä Forestry Field Station, University of Helsinki, Hyytiäläntie 124, FI-35500 Korkeakoski, Finland (H.K.); Arctic Research Center, Korea Polar Research Institute, 21990 Incheon, South Korea (J. Kim); Department of Atmospheric Sciences, University of Washington, Seattle, Washington 98195, USA (S.S.); Institute for Aerosol and Sensor Technology, University of Applied Science Northwestern Switzerland, 5210 Windisch, Switzerland (E.W.)., ,

**Keywords:** Atmospheric chemistry, Atmospheric chemistry, Thermodynamics

## Abstract

About half of present-day cloud condensation nuclei originate from atmospheric nucleation, frequently appearing as a burst of new particles near midday^1^. Atmospheric observations show that the growth rate of new particles often accelerates when the diameter of the particles is between one and ten nanometres^2,3^. In this critical size range, new particles are most likely to be lost by coagulation with pre-existing particles^4^, thereby failing to form new cloud condensation nuclei that are typically 50 to 100 nanometres across. Sulfuric acid vapour is often involved in nucleation but is too scarce to explain most subsequent growth^5,6^, leaving organic vapours as the most plausible alternative, at least in the planetary boundary layer^7,8,9,10^. Although recent studies^11,12,13^ predict that low-volatility organic vapours contribute during initial growth, direct evidence has been lacking. The accelerating growth may result from increased photolytic production of condensable organic species in the afternoon^2^, and the presence of a possible Kelvin (curvature) effect, which inhibits organic vapour condensation on the smallest particles (the nano-Köhler theory)^2,14^, has so far remained ambiguous. Here we present experiments performed in a large chamber under atmospheric conditions that investigate the role of organic vapours in the initial growth of nucleated organic particles in the absence of inorganic acids and bases such as sulfuric acid or ammonia and amines, respectively. Using data from the same set of experiments, it has been shown^15^ that organic vapours alone can drive nucleation. We focus on the growth of nucleated particles and find that the organic vapours that drive initial growth have extremely low volatilities (saturation concentration less than 10^−4.5^ micrograms per cubic metre). As the particles increase in size and the Kelvin barrier falls, subsequent growth is primarily due to more abundant organic vapours of slightly higher volatility (saturation concentrations of 10^−4.5^ to 10^−0.5^ micrograms per cubic metre). We present a particle growth model that quantitatively reproduces our measurements. Furthermore, we implement a parameterization of the first steps of growth in a global aerosol model and find that concentrations of atmospheric cloud concentration nuclei can change substantially in response, that is, by up to 50 per cent in comparison with previously assumed growth rate parameterizations.

**Supplementary information:**

The online version of this article (doi:10.1038/nature18271) contains supplementary material, which is available to authorized users.

## Abstract (web summary)

The growth of nucleated organic particles has been investigated in controlled laboratory experiments under atmospheric conditions; initial growth is driven by organic vapours of extremely low volatility, and accelerated by more abundant vapours of slightly higher volatility, leading to markedly different modelled concentrations of atmospheric cloud condensation nuclei when this growth mechanism is taken into account.

**Supplementary information:**

The online version of this article (doi:10.1038/nature18271) contains supplementary material, which is available to authorized users.

## Aerosol particle formation in clean air

The effect of atmospheric aerosols on clouds and the radiative forcing of the climate system remains poorly understood. It is thought that nucleation of aerosol particles from atmospheric vapours rarely proceeds in the absence of sulfuric acid. Now two papers in this week’s *Nature* point to a previously unappreciated role for highly oxygenated molecules (HOMs) in promoting new particle formation and growth, essentially a mechanism that produces aerosols in the absence of pollution. Jasper Kirkby *et al*. show that aerosol particles can form as a result of ion-induced nucleation of HOMs in the absence of sulfuric acid under conditions relevant to the atmosphere in the CLOUD chamber at CERN. Jasmin Tröstl *et al*. examined the role of organic vapours in the initial growth of nucleated organic particles in the absence of sulfuric acid in the CERN CLOUD chamber under atmospheric conditions. They find that the organic vapours driving initial growth have extremely low volatilities. With increasing particle size, subsequent growth is primarily due to more abundant organic vapours of slightly higher volatility.

**Supplementary information:**

The online version of this article (doi:10.1038/nature18271) contains supplementary material, which is available to authorized users.

## Main

**Extended Data Figure 1:**
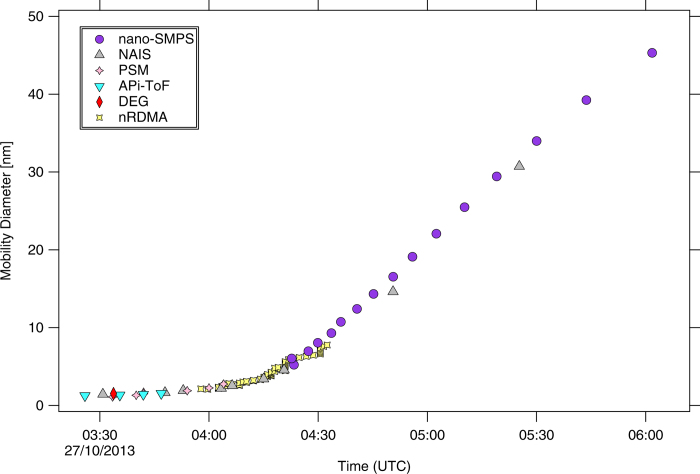
Appearance times of clusters and aerosols as seen by nano-SMPS, NAIS, PSM, APi-TOF, DEG and nRDMA. The different instruments are indicated with different plotting symbols. Instrument descriptions and acronyms can be found in Methods.

**Extended Data Figure 2:**
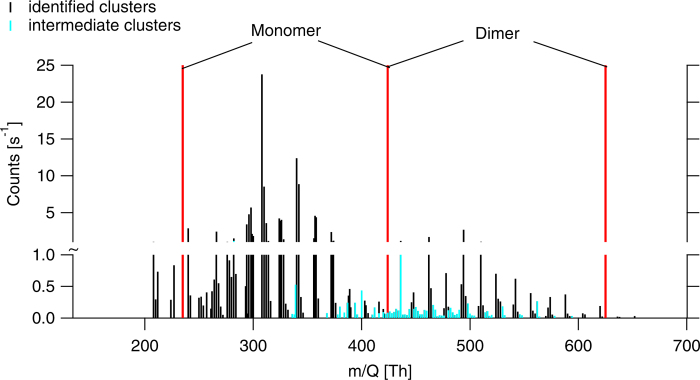
Observed mass spectrum as seen by the nitrate-CI-APi-TOF at 278 K and 38% relative humidity. A steady-state mixing ratio of approximately 250 p.p.t.v. of α-pinene was established in the chamber in the presence of 35 p.p.b.v. ozone and no injection of SO_2_. Black bars indicate all identified monomers and dimers, with the red bars indicating the corresponding *m*/*Q* range. Intermediate molecules or clusters (with carbon atoms between 11 and 17) that cannot be explained by the formation mechanism shown in Kirkby *et al*.^15^ are indicated by the cyan bars.

Two measurement campaigns at the CERN CLOUD (Cosmics Leaving OUtdoor Droplets) chamber (Methods) focused on aerosol growth with different levels of sulfuric acid and α-pinene oxidation products. With the chamber at 278 K and 38% relative humidity, tropospheric concentrations of α-pinene, ozone (O_3_) and SO_2_ were introduced (see Extended Data Table 1). Using various instruments (Methods and Extended Data Fig. 1) we measured the behaviour of freshly nucleated particles of 1–2 nm diameter and their subsequent growth up to 80 nm. Two chemical ionization mass spectrometers (Methods) using nitrate as the reagent ion (nitrate-CI-APi-TOF) measured the concentrations of sulfuric acid and highly oxygenated organic compounds^16,17^. Nitrate anions tend to cluster with highly oxygenated molecules (HOMs), and the measured HOMs fall broadly into two product ranges based on carbon number (Extended Data Fig. 2): monomers (C_*x*_H_*y*_O_*z*_ with *x* = 8–10, *y* = 12–16 and *z* = 6–12), and dimers (C_*x*_H_*y*_O_*z*_ with *x* = 17–20, *y* = 26–32 and *z* = 8–18). Here we refer to these measured compounds as HOMs rather than extremely low-volatility organic compounds (ELVOCs), as previously reported^17^. As we shall show, the HOM volatility spans a wide range (although it is always very low), and we shall separate HOMs into volatility bins using the volatility basis set (VBS)^18^.

**Figure 1:**
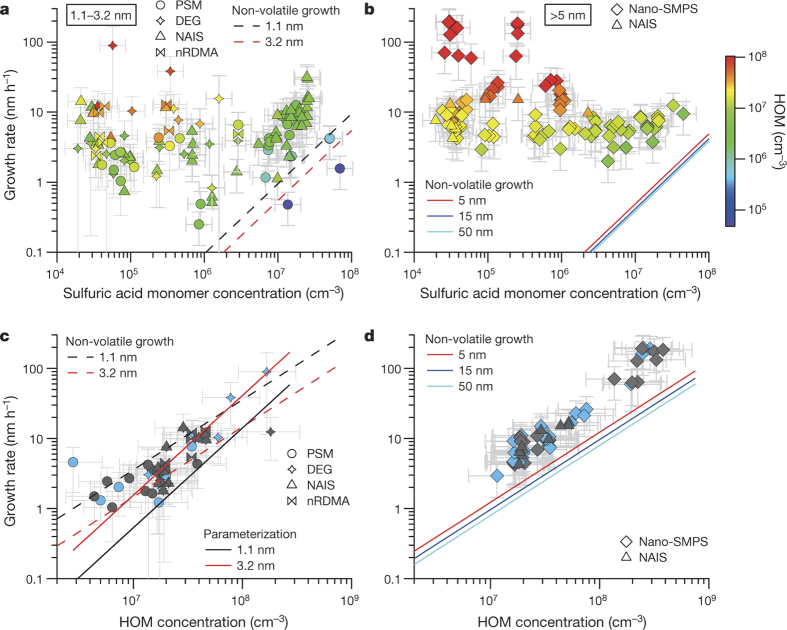
Growth rates as a function of sulfuric acid and highly oxygenated molecule (HOM) concentrations. Symbol shapes represent the different instruments to derive the growth rates (see key and Methods), symbol colours indicate the HOM concentration (colour scale at right). **a**, **b**, Growth rate versus sulfuric acid concentration for particles from 1.1 nm to 3.2 nm (**a**), and for particles 5–15 nm, 15–30 nm, 30–60 nm and >60 nm (**b**). Non-volatile growth rates by condensation of sulfuric acid^5^ are displayed for different diameters. **c**, Measured growth rates from 1.1 nm to 3.2 nm versus the HOM concentration for sulfuric acid concentrations <5 × 10^5^ cm^−3^; **d**, as **c** but for size ranges 5–15 nm, 15–30 nm, 30–60 nm and >60 nm. Linear growth was observed for particles >5 nm, thus no differentiation was made in **b** and **d**. Panel **c** additionally shows the parameterization for 1.1 nm and 3.2 nm based on our volatility-distribution modelling results. Symbol colours in **c** and **d** indicate the ion conditions in the chamber (blue, neutral; grey, ions from Galactic cosmic rays (GCR); see Methods). The HOM and sulfuric acid concentration uncertainty (error bars) is estimated to be +80%/−45% and +50%/−33%, respectively. Growth rate error bars indicate 1*σ* total errors. PowerPoint slide Source data

In Fig. 1 we plot the growth rates measured in CLOUD as a function of sulfuric acid and HOM concentration, focusing on size ranges from 1.1 nm to 3.2 nm and >5 nm (mobility) diameter. It is evident from Fig. 1a and b that the observed growth cannot be explained in either size range by the condensation of sulfuric acid even at the kinetic limit, where sulfuric acid is assumed to be completely non-volatile. Furthermore, for sulfuric acid molecular concentrations below 10^7^ cm^−3^, the growth rate is uncorrelated with sulfuric acid. In contrast, the growth is clearly correlated with organics for all size ranges up to the size of cloud condensation nuclei (CCN) for HOM concentrations >10^6^ cm^−3^ (Fig. 1c and d). For experiments with sulfuric acid concentration <5.5 × 10^5^ cm^−3^ we have separately reported a large charge enhancement for the nucleation rate^15^. However, there is no corresponding charge influence on the growth rates of either 1.1–3.2 nm or >5 nm particles (grey versus blue symbols in Fig. 1c and 1d). Most of the HOMs in the chamber are neutral (~10^7^ cm^−3^ neutral HOMs versus ~10^3^ cm^−3^ charged molecules), so a charge enhancement is not expected, especially with increasing size^19^. However, owing to the experimental uncertainties we cannot exclude the possibility of an ion enhancement at sizes below 3 nm.

A non-volatile (collision-limited) model of HOM condensation (Methods) cannot explain the observed growth rates across the full range of particle diameters we studied. We modelled growth at 1.1 nm, 3.2 nm, 5 nm, 15 nm and 50 nm (labelled curves, Fig. 1c and d) assuming that observed HOM monomers and dimers are non-volatile, with a density of 1,400 kg m^−3^ and a mass of 300 Da. Contrary to the common misconception that non-volatile diameter growth rate should be constant with size (in the free molecular regime), the predicted growth rate with this assumption is highest at any given HOM concentration for the smallest particles and decreases rapidly with increasing size up to ~5 nm (Fig. 1c, d). This predicted decreasing growth rate with increasing particle size is because the cross-section and collision velocity are highest relative to particle size for the smallest particles (Methods). However, the observations show the reverse, with growth rates for sizes above 5 nm exceeding those near 2 nm by a factor of 1.5 ± 0.2, obtained from normalizing (to 10^7^ cm^−3^ HOMs) and averaging the growth rates in the considered size ranges. The ratio of observed growth rates to modelled non-volatile growth rates increases from 0.7 ± 0.1 at 1.1 nm to 2.8 ± 0.2 at 5 nm, where in each case the quoted error is the standard error of the mean. This large discrepancy is strong evidence that the measured HOMs cannot fully describe the observed growth, and that additional organic material must be contributing to particle growth above roughly 5 nm particle diameter.

**Figure 2:**
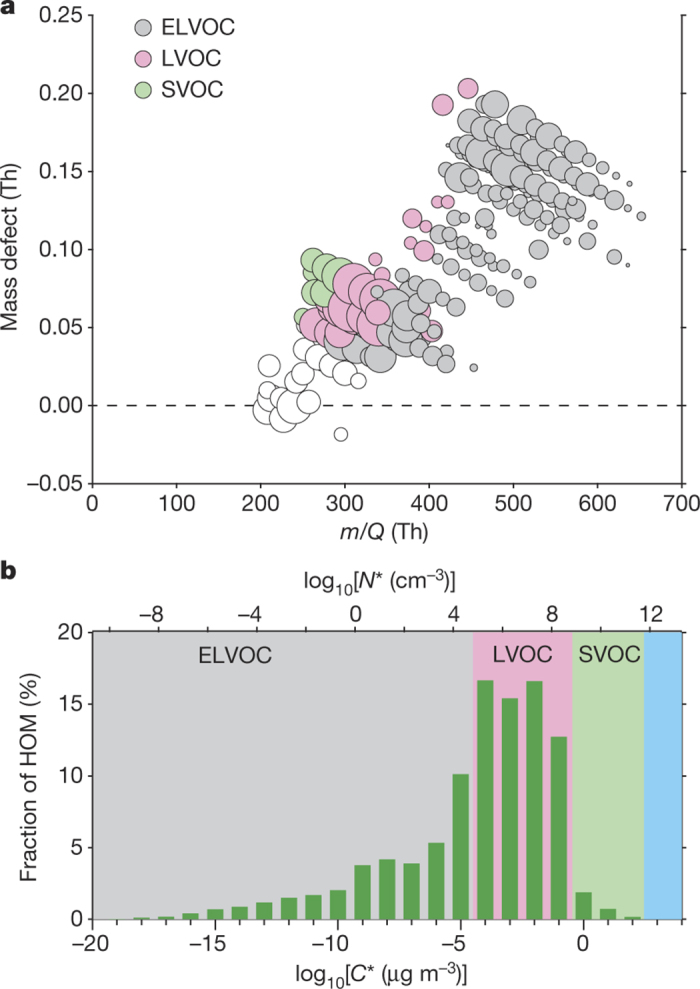
Observed gas-phase HOMs and their volatility distribution. **a**, Mass defect (in Th; 1 Th = 1 Da/*e* , where *e* is the elementary charge) of all HOMs versus their nominal mass to charge ratio (*m*/*Q*) including the estimated volatility distribution based on the proposed structures (Extended Data Fig. 3). The size of the plotting symbols is proportional to the logarithm of the counting rate. White circles are C_5_–C_7_ compounds, which were not included in the volatility analysis. **b**, HOMs binned to a volatility distribution showing the measured relative counting rates in per cent, with ELVOCs comprising ~36%. PowerPoint slide Source data

**Extended Data Figure 3:**
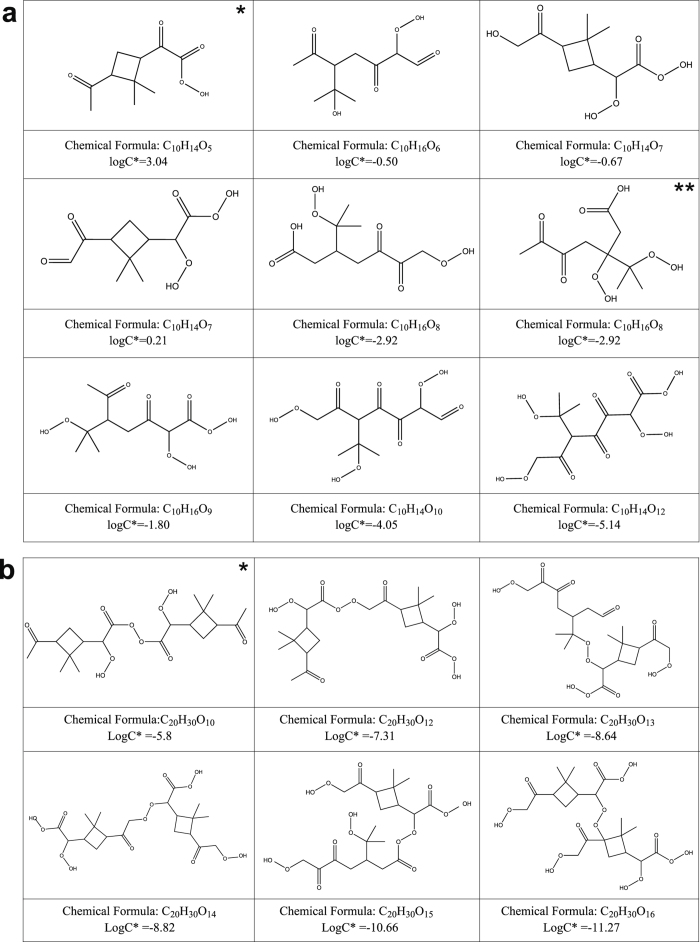
Possible structures of α-pinene oxidation products. **a**, Possible structures of HOM monomer molecules. C* was estimated using the SIMPOL method (at 293 K). Note that the volatility is less once the ring structure is open. The volatility generally decreases with increasing oxidation and decreasing temperature. **b**, Possible structures of HOM dimer molecules. C* was estimated using the SIMPOL method (at 293 K). Structures in boxes with asterisk(s) at the top right corner were confirmed by (*) or taken from (**) Kurtén *et al*.^74^.

**Extended Data Figure 4:**
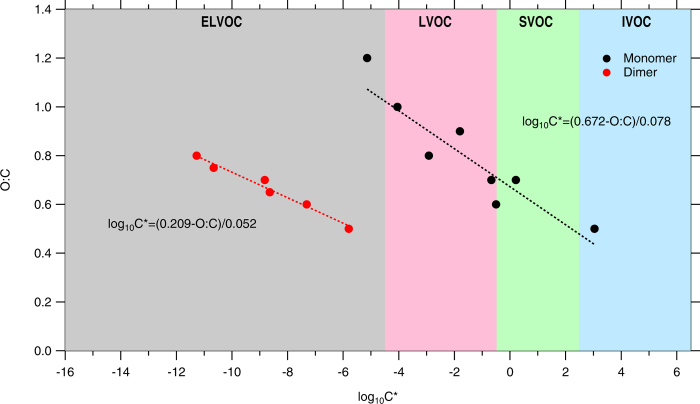
Estimation of C* for monomer and dimer molecules at 293 K. Colours indicate the volatility class based on ref. 18. A linear fit was applied to the C* estimates (dashed lines). This fit was then applied to all compounds using their O:C ratio to estimate their value of C*. Volatility bins comprise ELVOC, LVOC, SVOC and IVOC (intermediate volatile organic compounds with C* from 10^2.5^ to 10^6.5^ μg m^−3^).

To explore the potential role of HOM volatility, we use the SIMPOL model^20^ to estimate the saturation mass concentration (C*, μg m^−3^) and saturation molecular concentration (N*, cm^−3^) of each HOM using its measured atomic composition together with an estimation of its likely chemical structure (see Extended Data Fig. 3). We grouped the HOMs in volatility bins (separated by factors of ten) and assigned them to several volatility classes (see Extended Data Fig. 4). The HOMs span a wide range from extremely low-volatility (ELVOC, C* < 10^−4.5^ μg m^−3;^ N* < 5 × 10^4^ cm^−3^ assuming a molecular mass of 300 Da) to low-volatility (LVOC, 10^−4.5^ μg m^−3^ ≤ C* ≤ 10^−0.5^ μg m^−3^; 5 × 10^4^ cm^−3^ ≤ N* ≤ 5 × 10^8^ cm^−3^) to some semi-volatile (SVOC, 10^−0.5^ μg m^−3^ ≤ C* ≤ 10^2.5^ μg m^−3^; 5 × 10^8^ cm^−3^ ≤ N* ≤ 5 × 10^11^ cm^−3^) organic compounds. In Fig. 2a we show a mass defect plot (Methods) of the observed compounds during a representative run, and in Fig. 2b we show the corresponding volatility distribution (colours based on ref. 18). The binned volatility distribution of measured gas-phase organic species (Fig. 2b) shows a substantial fraction of ELVOCs, maximal contribution in the LVOC range and even low levels of SVOCs. Because the LVOCs and SVOCs do not build up a sufficient saturation ratio to overcome the Kelvin barrier, they should not be able to condense onto the smallest particles, so that only the ELVOCs should contribute to the initial growth. While nitrate ions cluster efficiently with ELVOCs and calibration based on sulfuric acid should be fairly accurate, the concentration of LVOCs and SVOCs is likely to be underestimated because of inefficient clustering^21^. Indeed, SVOCs are formed with high yield in α-pinene oxidation^22^ but most of them evidently are not detected by the nitrate-CI-APi-TOF instrument (Fig. 2). The fact that even the non-volatile model based on measured HOMs underestimates the observed growth rates for particles >5 nm by a factor of three strongly indicates that the concentration of condensing organic vapours is substantially higher than measured, at least after the Kelvin barrier has diminished.

**Figure 3:**
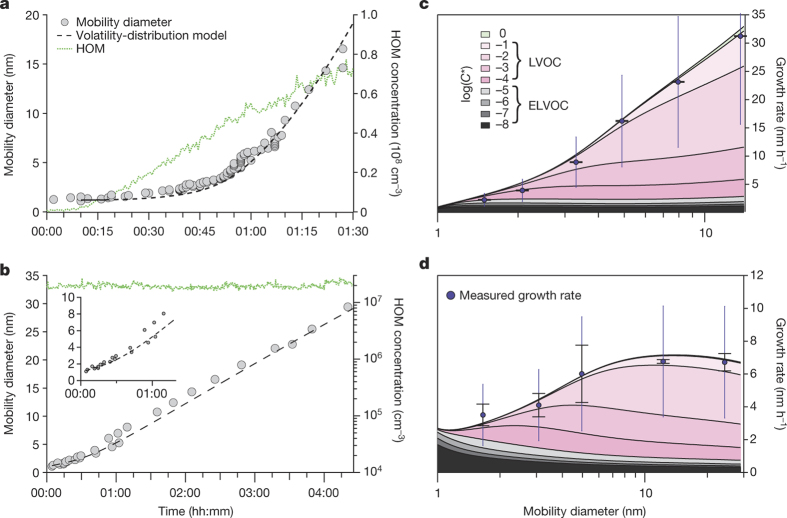
Comparison of the growth rates in two experiments with a dynamic volatility basis set (VBS) model. **a**, **b**, Temporal evolution of the particle size (filled circles) and the modelled particle size (dashed lines) for an experiment with increasing HOM concentration (**a**), and for constant HOM concentration (**b**), with the inset magnifying the time evolution of the first 5 nm. **c**, **d**, Size-dependent modelled (lines) and measured (filled circles) growth rate for the increasing HOM concentration (**c**), and for the constant HOM concentration (**d**). Colours (key in **c**) indicate the contribution of different volatility bins to the condensational growth. Error bars indicate the error of the fit alone, whiskers the 1*σ* systematic scale uncertainty of the determined growth rates. PowerPoint slide Source data

We further consider two very different experiments. During the first experiment, the HOM concentration increased nonlinearly with time, which replicates the diurnal variation of biogenic emissions and oxidants in the ambient for the morning and early afternoon (Fig. 3a). This situation leads to a nonlinear increase in the growth rate. During the second experiment, the HOM concentration remained at a constant steady state (production balanced by wall loss). This allowed us to test whether the accelerating growth seen in the first experiment was due to the diminishing Kelvin effect or the increasing HOM concentration. The constant HOM concentration led to a nearly constant growth rate, except for the smallest particles below ~5 nm (Fig. 3d).

**Extended Data Figure 5:**
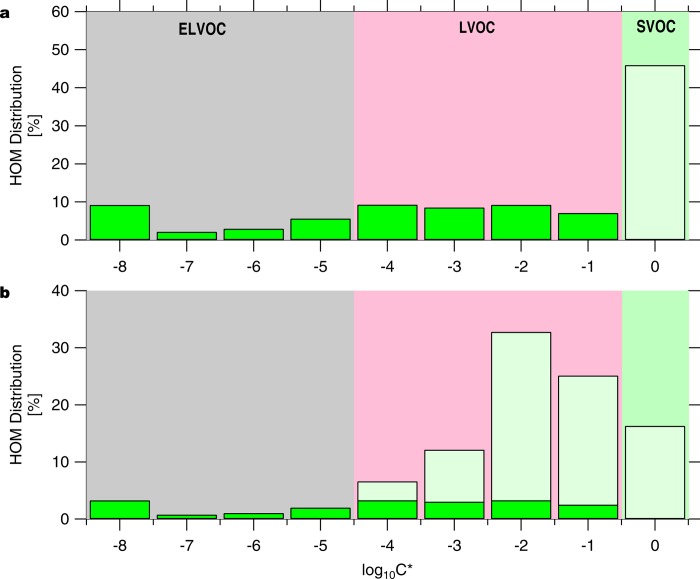
HOM distribution binned to a VBS. **a**, Measured HOM distribution (dark green) binned to a VBS. As the nitrate-CI-APi-TOF is expected to underestimate SVOC, which are often observed during secondary aerosol formation in smog chamber studies, we added a representative SOA bin at logC* = 0 (light green). **b**, Modified HOM distribution after scaling for the weaker charging efficiency for LVOC (light green). The ELVOC:LVOC:SVOC ratios are **a**, 20:34:46 and **b**, 7:77:16.

**Extended Data Figure 6:**
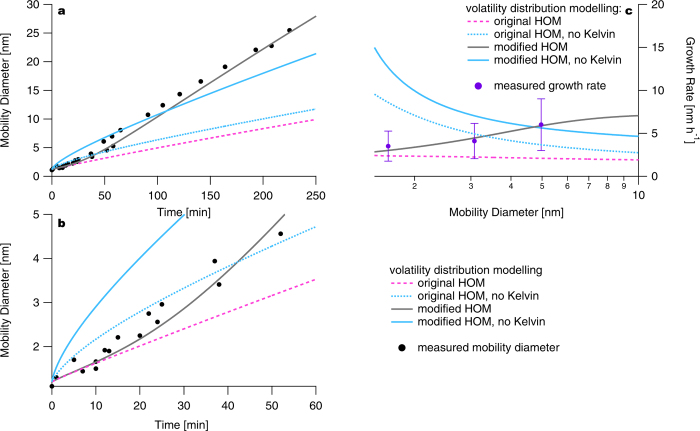
Dynamic volatility-distribution modelling results with and without a Kelvin term and with original and modified HOM volatility distribution for the case of constant HOMs. **a**, Different model approaches (key at bottom right) compared to the measured diameter evolution. **b**, Enlargement of the first 30 min of the experiment and the first 5 nm of the diameter evolution (key in panel). **c**, Size dependent growth rate for different model approaches (key at bottom right). The Kelvin effect is essential to describe the measured diameter behaviour. Using the original volatility distribution (blue dashed line), the model slightly overestimates the initial growth but strongly underestimates it at larger sizes. Although considering a Kelvin effect fits the initial growth well, growth at larger sizes is underestimated even more (pink dashed line). By adjusting the HOM volatility distribution in the model with no Kelvin effect, the best fit (blue solid curve) still fails to reproduce the observations, substantially overpredicting growth at small sizes and then underpredicting growth at larger sizes. However, adjusting the volatility distribution and treating the Kelvin effect captures the growth well over the full size range (grey solid line). Error bars indicate the 1*σ* systematic scale uncertainty of the determined growth rates.

**Extended Data Figure 7:**
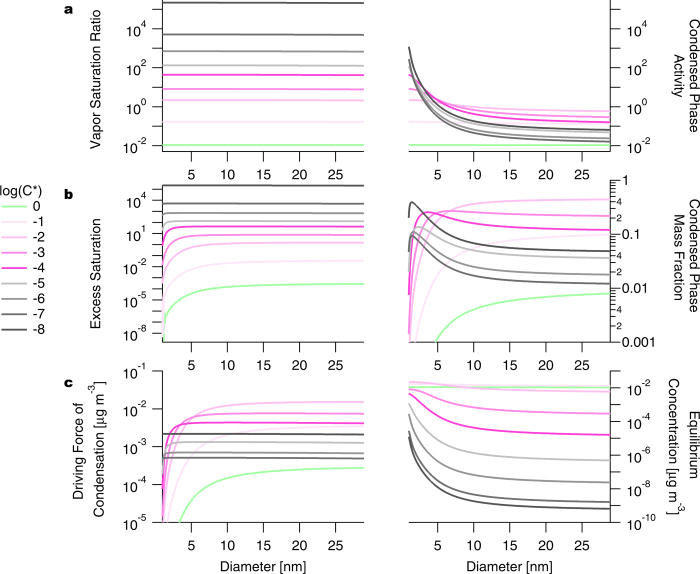
Dynamic volatility-distribution model details. **a**, Vapour (left) and condensed-phase (right) activities during a simulated particle growth event in CLOUD (Fig. 3b, d). Vapours are in steady-state with respect to production and wall loss, with the saturation ratio increasing monotonically with decreasing volatility. **b**, Excess saturation ratios (left) and particle composition (mass fractions; right) during simulated particle growth event in CLOUD. **c**, Driving force of condensation (left) and equilibrium concentrations of vapours over particles (right) during simulated particle growth events in CLOUD. Volatility is indicated by brightness, with darkest grey corresponding to C* = 10^−8^ μg m^−3^ (see key).

In order to quantify the importance of the Kelvin effect and HOM measurement biases, we analysed the contribution of HOMs to early growth and assessed the dependence on HOM volatility by using a dynamic volatility-distribution model^23^ for these two cases. The HOM volatility-distribution model comprises nine C* bins ranging from 10^−8^ μg m^−3^ to 1 μg m^−3^ (10^1^ cm^−3^ to 10^9^ cm^−3^), split into three ranges (see Fig. 2 and Extended Data Fig. 5): ELVOC (grey), LVOC (pink) and SVOC (light green). When we run the HOM volatility-distribution model using the directly measured volatility-binned HOM concentrations as input, the simulated growth rates for particles >2 nm are underestimated by a large factor (see Extended Data Fig. 6, blue dashed line). This is consistent with the expectation that the detection efficiency of LVOCs in the nitrate-CI-APi-TOF is lower as discussed above. An attempt to adjust the HOM volatility distribution by increasing the LVOCs to reproduce the observed growth rates was not successful (see Extended Data Fig. 6, blue solid line). The model can be brought into agreement with observations by increasing the LVOC concentrations and introducing a Kelvin effect (Fig. 3 and Extended Data Fig. 6 grey line). This tuned model, adjusting for inefficient LVOC measurement in the nitrate-CI-APi-TOF and considering the Kelvin effect (see Methods, Extended Data Fig. 5b and Extended Data Fig. 7 for details), captures the observed particle growth in both example cases with high fidelity (Fig. 3). While the agreement at 10 nm diameter is ensured by our LVOC correction, the Kelvin term is essential to reproduce the observed growth rate over the full size range for these two quite different cases, although the strong size dependence in Fig. 3a is primarily due to the increasing HOM concentration. This is evidence that the Kelvin term (along with abundant LVOCs) is responsible for the acceleration in growth observed in field experiments in the afternoon, and that only ELVOCs have a sufficiently high saturation ratio to overcome the Kelvin barrier at the smallest sizes.

**Extended Data Figure 8:**
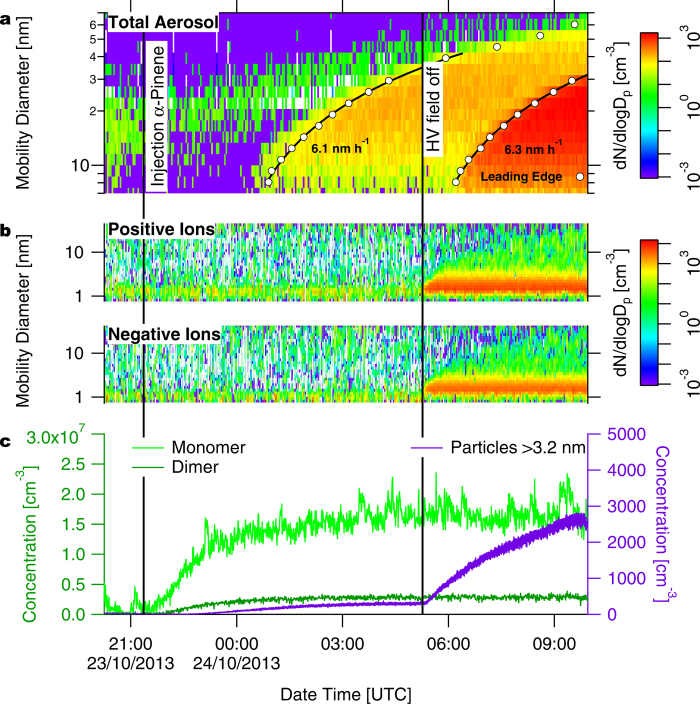
Typical experiment during CLOUD8. α-Pinene was injected under neutral conditions. Once stable conditions were achieved, the clearing (HV) field was turned off allowing ions from Galactic cosmic rays to remain in the chamber. This immediately yields a second nucleation. **a**, The nano-SMPS size distribution; **b**, the ion size distribution as seen by the NAIS ion mode; and **c**, the monomer (light green) and dimer (dark green) and the number particle concentration for particles bigger than 3.2 nm (purple; CPC 3776). The colour keys on the right side relate to the number size distribution (d*N*/dlog*D*_p_).

The pool of ELVOCs, many having  μg m^−3^ (Fig. 2b), implies continuous production of relatively stable clusters smaller than 2 nm (continuous nucleation is observed, as shown in Extended Data Fig. 8). ELVOCs govern the contribution to growth up to ~2 nm; beyond this, LVOCs take over in sequence as the Kelvin effect becomes progressively weaker with increasing size. Thus, while growth rates in the non-volatile HOM model decrease by a factor of ~3 between 1 nm and 5 nm, in the volatility-distribution HOM model they increase by a factor of ~3 over this range, consistent with observations. This volatility-distribution growth model is a version of ‘nano-Köhler theory’, in which the effects of condensed-phase mixing (Raoult’s law) and particle curvature (the Kelvin term) combine for miscible organics. The Kelvin effect dominates because curvature enhances condensed-phase activities by orders of magnitude for the smallest particles, regardless of their composition, and the critical issue is whether the saturation ratio of an LVOC volatility bin exceeds this threshold (see Extended Data Fig. 7 for detailed model results). Finally, the volatility-distribution model shows that, in the experiments, SVOCs cannot contribute to the observed growth via non-reactive uptake as their gas-phase saturation ratio never rises high enough for them to contribute (see Extended Data Fig. 7 and Methods).

The α-pinene + ozone system explored here is among the most efficient sources of ELVOCs yet observed^16,17^, but it is likely that many sources of LVOCs may be important in the atmosphere. The latter include the first-generation compounds described here but also later-generation ‘ageing’ products formed by reaction with OH radicals^10,24,25^. Different sources are almost certain to produce LVOCs with differing volatility distributions and chemical properties, which also might influence their reactivity in the condensed phase, including oligomerization^23^ and reactive uptake^26^, resulting in different growth patterns compared to those in Fig. 3. These growth patterns thus constitute a critical and variable link between new particle formation and CCN formation.

**Figure 4:**
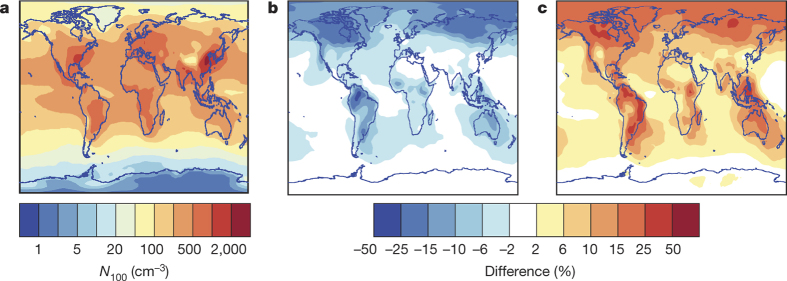
Modelled influence on global CCN of different organic growth rates from 1.7 nm to 3 nm simulated by the GLOMAP aerosol model. **a**, The annual mean number concentration of soluble particles of at least 100 nm diameter (*N*_100_ on colour scale, taken as a proxy for CCN) at cloud base level. We treat irreversible (collision-limited) condensation of sulfuric acid for particle growth from 1.7 nm to 3 nm, together with a size-dependent growth rate due to HOMs from the present work. **b**, The percentage change in CCN concentration (colour scale) when growth from 1.7 nm to 3 nm is due to sulfuric acid alone. **c**, The percentage change in CCN concentration when we parameterize growth from 1.7 nm to 3 nm as irreversible condensation of sulfuric acid together with an organic contribution following ref. 30, which assumes a Kelvin barrier to organic condensation below 2.5 nm. All simulations assume the same nucleation rates at 1.7 nm and the same particle growth rates above 3 nm. PowerPoint slide

**Extended Data Figure 9:**
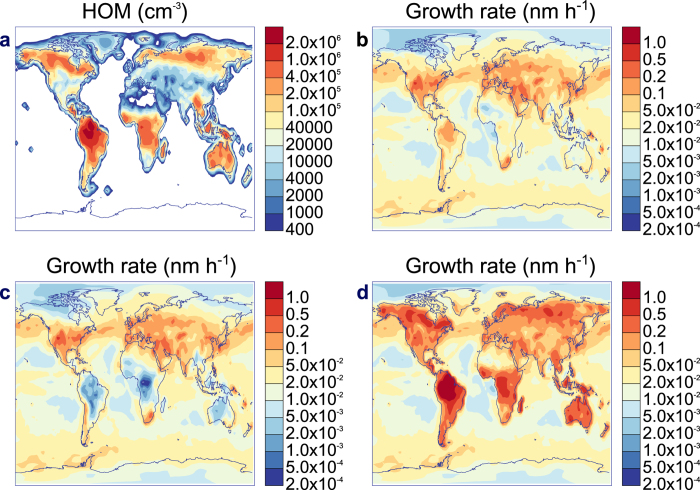
Annually averaged HOM concentration, and the annually averaged growth rate, simulated by GLOMAP at cloud base level. **a**, Spatial distribution of HOM concentration (in cm^−3^). **b**–**d**, Spatial distribution of growth rates (in nm h^−1^) using different parameterizations: **b**, using the size-dependent parameterization of initial particle growth and irreversible condensation of H_2_SO_4_, **c**, with growth from 1.7 nm to 3 nm only due to H_2_SO_4_, and **d**, with growth from 1.7 nm to 3 nm assuming irreversible condensation of H_2_SO_4_ together with an organic contribution following ref. 30, which assumes a Kelvin barrier to organic condensation below 2.5 nm.

Strongly size-dependent nanoparticle growth has been observed and parameterized based on atmospheric observations^3,27,28,29^, although during nucleation events in the field it has not been possible to determine whether changes in the growth rate are due to the Kelvin effect or due to changes in the HOM concentrations during the event. To assess the global implications of our findings, we parameterized the growth between 1.7 nm and 3 nm using the size-resolved growth rates from the HOM volatility-distribution modelling results (Fig. 1 and Methods). Using a global aerosol model (Methods), we find that CCN concentrations are sensitive to whether, and how, organic compounds participate in the first stages of the growth of freshly nucleated particles. Figure 4a shows the concentrations of soluble 100 nm particles (*N*_100_), a proxy for CCN, using our parameterized growth rates, which are up to a factor of two higher than those in a simulation without organics participating in the initial growth (Fig. 4b). Conversely, a previous parameterization^30^ which empirically accounts for the Kelvin effect below 2.5 nm but assumes that all condensable organic products (not just HOMs) contribute to the growth of these particles, produces CCN concentrations up to 50% higher than our parameterization (Fig. 4c). Our model results show that CCN concentrations can be sensitive to the processes and concentrations of species driving the growth of the smallest atmospheric particles as reflected in the pronounced differences of the corresponding growth rates (Extended Data Fig. 9). On the basis of the combined modelling results and experimental data that we report here, we suggest that low-volatility organic vapours are the key to particle growth at the initial sizes.

## Methods

### The CLOUD chamber

We conducted two measurement campaigns at the CERN CLOUD chamber, a 26 m^3^ stainless steel vessel which enables aerosol experiments under the full range of tropospheric conditions^31,32^. CLOUD7, in the autumn of 2012, included mostly high sulfuric acid concentrations, while CLOUD8, in 2013, addressed low sulfuric acid concentrations. To avoid contamination, pure air is generated by the evaporation of cryogenic liquid nitrogen (N_2_) and liquid oxygen (O_2_), combined at a ratio of 79:21. A UV light (250–400 nm) system enables the formation of hydroxyl (OH) radicals via the photolysis of ozone^33^. By applying a high voltage field (30 kV m^−1^) all charged particles in the chamber can be removed rapidly (neutral conditions); when the high voltage field is turned off, natural ions are produced in the chamber by Galactic cosmic rays (GCR condition) reaching ground level. With the 3.5 GeV/*c* secondary pion beam (*π* condition) from the CERN Proton Synchrotron passing through the chamber, ion concentrations representative for those of the upper troposphere can be achieved^31,34^. A dedicated inlet system is available for every gas. In order to clean the chamber, the chamber can be heated by raising the temperature to 373 K, and, in addition, flushed with ultra pure water. All gas pipes are made from stainless steel to avoid contamination, and chamber and gas seals are chemically inert gold coated metal. Two fans running in counter flow ensure a good mixture of the gases in the chamber^35^. Traces of contaminants, for example, low molecular weight volatile organic compounds (VOCs)^36^ or ammonia^37^, were sometimes observed in the chamber. However, as shown elsewhere^36^, extremely clean conditions can be achieved.

### Experimental settings

A typical experiment started with the injection of α-pinene under neutral (ion free) conditions. The ozone already present in the chamber immediately reacts with the α-pinene leading to aerosol nucleation (see also ref. 15). Using the UV fibre system in the chamber, additional OH could be photochemically produced. The major fraction of HOM (~60%) were chemically produced via the ozonolysis of α-pinene. This experiment was continued until a steady-state—that is, a stable HOM concentration, was achieved. Afterwards the high-voltage field, used in neutral experiments for sweeping out ions, was turned off. This allowed ions (~700 cm^−3^) produced by Galactic cosmic rays to accumulate in the chamber, and resulted in a second nucleation event (see also ref. 15). In addition experiments were also started under GCR conditions to prove consistency. In total, approximately 40% of the runs started (with increasing HOM concentration) in neutral conditions, 18% in GCR condition and 20% in *π* condition. Plateau conditions (with steady-state HOM concentration) in GCR constitute approximately 18% of the runs and in *π* condition approximately 4%. *π* conditions relate to experiments where the Proton Synchrotron was also used to produce higher ion concentrations (~3,000 cm^−3^), as encountered in the upper troposphere. This was only possible during CLOUD 7, as during CLOUD 8 the Proton Synchrotron was not in operation due to maintenance work. A typical experiment is shown in Extended Data Fig. 8. For pure biogenic experiments, we added no SO_2_; for sulfuric acid experiments, we injected SO_2_ into the chamber as an additional precursor. All experimental steady-state conditions can be found in Extended Data Table 1. For each run several growth rates at different diameters could be quantified (see Extended Data Figs 1 and 8). Extended Data Fig. 8 shows two nucleation events that were observed during one run, one under neutral and the second one under GCR conditions. Thus, one run can yield several points in Fig. 1.

### Cluster composition

*Atmospheric pressure interface time of flight mass spectrometer (APi-TOF).* One APi-TOF (Tofwerk AG) measured the mass-to-charge ratio of positive or negative clusters present in the CLOUD chamber^24^. Since this instrument only measures charged clusters, the measurements were made during GCR or *π* conditions. It is only possible to measure one polarity at a time thus positive and negative spectra were measured alternately.

*Chemical ionization atmospheric pressure interface time of flight mass spectrometer (nitrate-CI-APi-TOF).* Two nitrate-CI-APi-TOFs^38^ measured the concentration of sulfuric acid, oxidized organics and other clusters and molecules in the cloud chamber.

The instruments use an ion source (one an X-ray generator, one a corona needle) to ionize the reagent gas nitric acid in a nitrogen flow. In a drift tube an electric field is then applied to guide the primary ions to the sample flow where they react with the neutral molecules and clusters with an overall reaction time of about 200 ms in one instrument and 50 ms in the other. Inside the drift tube, two possible reactions can then take place to ionize the neutral molecules or clusters A in the sample flow:The first reaction (R1) corresponds to a proton transfer reaction (acid/base reaction) which is, for example, the case for sulfuric acid. The second reaction (R2) is a ligand switching reaction, forming a more stable adduct, which is the case for highly oxygenated molecules (HOMs). Using an electrostatic field, the charged molecules and clusters are then guided through a small pinhole with a diameter of 350 (300) μm to the APi-TOF section.

The voltage settings in the APi and TOF sections determine the mass dependent transmission efficiency of the instrument. The transmission curves were determined with separate measurements, by adding certain compounds (perfluorinated acids) to the instrument in sufficient amounts to deplete the primary ions. With this method the transmission relative to the mass to charge ratio (*m*/*Q*) of the primary ions was determined^39^. One instrument operated at the same voltage settings for the whole campaign while the other one was operated in a switching mode between voltage settings optimized for a low or high *m*/*Q* range.

We analysed the raw data with the MATLAB *tofTools* package 32^40^. The mass scale is calibrated to better than 10 p.p.m. accuracy, using a two-parameter fit. The concentration of sulfuric acid is calculated from the count rates of each ion species as follows:where [H_2_SO_4_] is the concentration of sulfuric acid. The corresponding ion count rates, including the primary ions, appear on the right hand side of the equation. *C* is a calibration coefficient, which was determined by connecting the nitrate-CI-APi-TOF to a well characterized H_2_SO_4_ generator^41^. Line losses for H_2_SO_4_ were corrected with the term . SL can be calculated from empirical equations for straight circular tubes with a laminar flow^42^.

### Measurement of oxidized organics

During nucleation and growth, we observed two distinct signal patterns—monomers and dimers—in the nitrate-CI-APi-TOF (Extended Data Fig. 2, Run 1209) corresponding to the monomers and dimers of the α-pinene oxidation products. These bands contain highly oxygenated molecules (HOMs), which have been found to play a potentially major role in aerosol growth^17^. Owing to their structure and their high O:C, these clusters have a low saturation vapour concentration. In ref. 17, it was assumed that all observed oxygenated organics are extremely low-volatility organic compounds (ELVOCs) and condense on the added seed aerosol.

We define monomers (mainly C_*x*_H_*y*_O_*z*_ with *x* = 8–10, *y* = 12–16 and *z* = 6–12) as the sum of the peaks in the *m*/*Q* range from 235–424 Th (1 Th = 1 Da/*e*, where *e* is the elementary charge) and dimers (mainly C_*x*_H_*y*_O_*z*_ with *x* = 17–20, *y* = 26–32 and *z* = 8–18) as the sum from 425–625 Th. We excluded contamination peaks from the summation within a band, as well as peaks assigned to RO_2_ radicals (C_10_H_15_O_6,8,10,12_, corresponding to *m*/*Q* of 293, 325, 357 and 389 Th).

The APi-TOF also detected naturally charged clusters between 670 and 850 Th (trimers) and between 900 and 1,200 Th (tetramers). For the nitrate-CI-APi-TOF the trimer band was only observed for a very long integration time, indicating either a low concentration of neutral trimers or a low transmission efficiency. We also observed intermediate species with a carbon number of 11 to 17, which may be dimers formed from reactions of RO_2_ radicals with RO_2_ radicals formed from fragments. However, their concentration is small (see cyan peaks in Extended Data Fig. 2).

To estimate the concentration of each highly oxygenated molecule (HOM_*i*_), we applied the following equation:In this equation,  is the integrated area of a background corrected HOM peak in counts per second (c.p.s.). We corrected for the losses through the sampling line with the term SL_HOM_. Here, we used the diffusion coefficients for the monomers (0.0297 cm^2^ s^−1^) and for the dimers (0.0240 cm^2^ s^−1^), which we determined in the CLOUD chamber experimentally. This results in correction factors for the monomers of a factor of 1.44 and for dimers of a factor of 1.37. The total HOM concentration is defined as the sum of all [HOM_*i*_], which includes all identified monomers, dimers and intermediate clusters (see Extended Data Fig. 2).

We assume that the binding between the nitrate ion and the HOM is strong and proceeds at the kinetic limit and therefore use the same calibration constant *C* as for sulfuric acid. This assumption does hold for highly oxygenated species with extremely low volatilities, but not for less oxygenated species as the ionization efficiency decreases^21^. Quantum chemical calculations have shown that the nitrate preferably clusters with ELVOC^21^. Less oxidized species are, therefore, observed to a lesser extent under our experimental conditions (HNO_3_ concentration).

The transmission efficiency *T*_*i*_ of each individual HOM_*i*_ depends strongly on the mass of each molecule and the different voltage settings in the nitrate-CI-APi-TOF. To correct this transmission factor, we derived a transmission curve over the whole mass range of the HOMs. For more details see ref. 43.

The uncertainty in HOM measurement was caused by the following sources: uncertainty in sulfuric acid calibration, charging efficiency of HOMs by the nitrate ion, mass dependent transmission efficiency and sampling line losses. This results in an overall scaling uncertainty for the measured [ELVOC] of +80%/−45% assuming a charging efficiency of one. We cannot give an uncertainty of the LVOC concentration. Instead we used a scaling factor to match the observation. On the basis of that and because LVOC  ELVOC, the HOM concentration is presumably underestimated by a factor of four. Nobody, at least to our knowledge, has been able to calibrate the nitrate chemical ionization source for charging efficiency so far.

For the analysis, the data from only one nitrate-CI-APi-TOF (University of Frankfurt–UFRA) was used. The main reason for this was that a transmission calibration of the APi-TOF section was performed with this instrument (see also ref. 43) and thus the data are expected to be quantitatively correct. The other nitrate-CI-APi-TOF (University of Helsinki–UHEL) agrees very well for the monomer concentration, but less well for the oligomers. In addition, the UHEL nitrate-CI-APi-TOF was operated under different settings. It was switched between several modes—(1) high fragmentation, (2) high mass and (3) low mass—to get further information on the fragmentation of the molecules and clusters.

*Mass defect.* In a mass defect plot, the difference between the exact mass of a compound and its nominal mass (Th) is depicted as function of its mass to charge ratio (Th). Depending on the element the mass defect can be negative or positive. In case of oxygen the mass defect is negative, so that a slope downwards represents an increase in oxygen molecules. Thus, the analysis of a complex high resolution spectrum is simplified by a convenient visualization where the pattern of compounds belonging to the same family is clearly shown.

*Proton transfer reaction time of flight mass spectrometer (PTR-TOF-MS).* We used a PTR-TOF-MS (Ionicon Analytik) to determine α-pinene concentrations in the chamber; it also provides information about the overall cleanliness regarding VOCs in the chamber. VOCs are ionized in a reaction chamber by means of a proton transfer reaction under precisely defined conditions (reaction time, pressure, temperature) and then analysed by a time-of-flight (TOF) mass spectrometer (Tofwerk AG). A mass resolving power of 4,000 (*m*/∆*m*, FWHM) and a mass accuracy within 10 p.p.m. enables unambiguous identification of pure hydrocarbons and volatile organic compounds up to *m*/*Q* = 250 Th (ref. 39). Direct calibration allows determination of α-pinene volume mixing ratios with an accuracy of 5% and a lower detection limit of 25 parts per trillion by volume (p.p.t.v.).

*SO*_*2*_*chemical ionization mass spectrometer (SO*_*2*_*-CIMS)*. The very low SO_2_ volume mixing ratios were determined with an SO_2_ chemical ionization mass spectrometer (SO_2_-CIMS). It uses the primary ion  to convert SO_2_ to  (reaction scheme can be found elsewhere^44^). The  is then measured in a quadrupole mass spectrometer with an atmospheric pressure interface (Georgia Tech). The primary ions are generated with a corona discharge^45^. The ratio of  to  was maximized by feeding CO_2_, O_2_ and Ar directly over the corona discharge, leading to a reduced contamination by . The SO_2_ concentration is then calculated as follows:where *R*_112_ is the background-corrected ion count rate of , *R*_60_ the ion count rate of  and *C*_*s*_ the calibration factor. *C*_*s*_ was obtained by using an SO_2_ gas standard (Carbagas AG). The calibration was repeated periodically during the campaign. The resulting calibration factor was found to be 1.3 × 10^5^ p.p.t.v. Its detection threshold of SO_2_ is about 15 p.p.t.v.; the uncertainty is within 23% for low SO_2_ volume mixing ratios (around 30 p.p.t.v.), and 13% for volume mixing ratios >150 p.p.t.v. This uncertainty is mostly related to temperature changes in the experimental hall where the SO_2_-CIMS was located. This change led to a drift in the  background signal.

### Aerosol properties

*Nano radial differential mobility analyser (nRDMA).* A custom-built aerosol size classifier and counter assembly was used to measure positively charged particles in the 1.1 to 10 nm diameter size range with a time resolution of 60 s. The classifier was a Caltech Nano-Radial Differential Mobility Analyser (herein referred to as nRDMA^46^). The counter that was employed downstream of the nRDMA was an Airmodus Particle Size Magnifier with a 78 °C saturator coupled to a Brechtel Manufacturing Inc. Mixing Condensation Particle Counter, model 1710^47^. The raw data from the Caltech assembly was inverted using transfer function parameters, effective length, and penetration efficiency functions^48^.

*Nano scanning mobility particle sizer (nano-SMPS).* The nano-SMPS^49^ measured the dry aerosol size distribution from 5 nm to 80 nm with a time resolution of 130 s. It was located within a temperature controlled rack and was kept at chamber temperature. The nano-SMPS consisted of the TSI condensation particle counter (CPC) 3772 with a modified cut-off (*D*_50_ = 5.6 nm, *D*_10_ = 3.5 nm)^6^, a TSI-type PSI-built short differential mobility analyser (DMA) and a neutralizer (Kr-85 source). The data were corrected for single charging efficiency, multiple charges, diffusion losses, and CPC detection efficiency. The diffusion loss correction assumes a laminar flow^50^ and includes all parts of the nano-SMPS system (tubes, Kr-source, DMA inlet, DMA column).

*Neutral cluster and air ion spectrometer (NAIS).* The NAIS (Airel) is an ion mobility spectrometer designed to determine the number size distribution of ions in the size range 0.75–45 nm, as well as total (charged and neutral) particles in the size range ~2–45 nm (ref. 51). Previous studies have verified the performance of the NAIS^52,53^. It consists of two differential mobility analysers (DMAs) in parallel. Each is equipped with 21 electrometers, to separate the mobilities and determine the concentrations of positive and negative ions simultaneously. A corona charger is used when measuring the total particle size distribution.

*Particle counters.* Several particle counters with different 50% cut-offs were deployed at the CLOUD chamber including two DEG-CPCs^54,55^ (1.5 and 2.7 nm cut-off), one butanol CPC (TSI 3776, 3.2 nm cut-off) and one Particle Size Magnifier (PSM, Airmodus, model A10)^56^. The PSM was run in scanning mode and was used to determine the number size distributions between 1.4 nm and 3.4 nm mobility diameter.

### Volatility of oxygenated organics

Recent studies have focused on the formation mechanism of highly oxygenated organics^17,57,58^. Here we considered the propagation and termination reactions as proposed in refs 57 and 59. We used the radicals from α-pinene ozonolysis proposed in ref. 60 as a starting point and evaluated the possible chemical structures for monomers and dimers (Extended Data Fig. 3). We assume that dimers are covalently bound^15,17^. This is supported by the chemical formulae of the observed compounds which cannot be explained by a cluster consisting of two monomers.

Instead of assuming an average reduction of the saturation vapour concentration with oxidation, we used this set of chemical structures to calculate the saturation vapour concentration with SIMPOL^20^.

We then plotted the oxygen to carbon ratio (O:C) as a function of C* (see Extended Data Fig. 4). We applied a linear least squares fit and used the fit parameters to estimate the volatility for molecules for which we did not derive the structure. The intermediate cluster volatilities were roughly estimated assuming different numbers and types of functional groups (aldehydes, ketones, hydroperoxyacids). The concentration of these clusters is low and will therefore not influence the results significantly. SIMPOL was originally derived at 293 K, but a temperature dependence is given. Thus, we extrapolated C* to 278 K (resulting in approximately one order of magnitude lower C* values). Then we separated all observed HOM peaks into volatility regimes^18^, as shown in Fig. 2a and b. For this, the HOM concentrations observed in CLOUD for a steady-state run (1209) with ~600 p.p.t.v. of injected α-pinene was used. It needs to be noted that the SIMPOL data set does not contain the smallest saturation vapour pressures (as they are difficult to measure quantitatively). Thus, the predicted saturation vapour concentrations for low-volatility compounds could deviate from the actual values. However, the binned volatility distribution is rather flat especially in the ELVOC range. So even if the saturation concentration were to deviate by an order of magnitude, this would not change the conclusions of this work.

### Aerosol growth model

The net condensation flux is defined as^61^:with *N*_p_ the particle number concentration, *D*_p_ the particle diameter, *D*_*i*_ the vapour diameter, *α*_*i*,p_ the accommodation coefficient, the vapour concentration  and the saturation vapour concentration of . In the following the indicated terms of equation (4) will be further explained.

*Deposition rate coefficient.* In the molecular regime the collision cross-section is the appropriate metric of a collision probability. Here we assume hard-sphere limit, neglecting charge interactions. The deposition rate coefficient is corrected for the transition regime using the *β*_*i*,p_ correction factor, to account for non-continuum effects, that is^62^:The *β*_*i*,p_ correction term and the mass accommodation coefficient *α*_*i*,p_ are connected, as the correction term considers the onset of the gas-phase concentration gradients near the particle. For very small particles (Knudsen number, ), no gradients exist. However, for very large particles (), the gas concentration at the particle surface can be near zero even with *α*_*i*,p_ < 1. The effective mass accommodation coefficient, , is therefore introduced as well.

For the collision between vapours and ultrafine particles, the reduced mass *μ*_*i*,p_ needs to be considered; *v*_*i*,p_ is then the centre of mass velocity:The two first terms—collision cross-section and the deposition rate—can be combined. Instead of using the cross-section, the suspended surface area  can be used. The modified deposition rate coefficient is then given by:*Condensation sink.* Combining the surface area and the deposition rate coefficient we can calculate the collision frequency, which is the frequency with which species *i* collides with the particle surface:The condensation sink,  , gives the actual time constant for interaction of vapours with particles. The condensation sink is also the fundamental equilibration timescale between the gas and particle phases when condensation is the main loss of vapours.

*Driving force of condensation.* The driving force of condensation *F*_*i*,p_ and excess saturation ratio  are:The saturation ratio (gas-phase activity) is . The term *a*_*i*,p_ is the activity of the species *i* at the condensed-phase surface of the particle (*a*_*i*,p_ = *X*_*i*,p_*γ*_*i*,p_, Raoult term), where  is the mass fraction, and *γ*_*i*,p_ the mass based activity coefficient in the organic condensed phase. Owing to their curved surfaces, the activity of a small particle——includes the Kelvin term *K*_*i*,p_. The Kelvin term is defined as^61^:with the surface tension *σ*, the molar weight *M* and the density *ρ*. For very small particles a large supersaturation is needed to allow for condensation. For *σ* = 0.023 N m^−1^, a molar weight of 300 g mol^−1^ at 300 K, *D*_*K*_ = 3.75 nm. Any charge effect on the growth rate would appear in either an enhancement to the collision cross-section, *σ*_*i*,p_, due to charge-dipole interactions, or a change in the effective Kelvin diameter reflecting enhanced stability of small clusters. Further investigation of a possible enhancement in the growth rate caused by ions requires dedicated experiments.

*Equilibrium solution.* At equilibrium, *F*_*i*,p_ is zero. In this case, equilibrium partitioning is the basis for organic aerosol calculations. Aerosol partitioning theory describes the condensation and evaporation of gas phase species on or from an aerosol surface^63^. The fraction of the condensed phase (*s*) of a species *i* in the suspended aerosol particle within the partitioning frame work is defined as: is the effective saturation concentration of the vapour and  the concentration of species *k* in the particle phase.

*Steady-state solution.* Organic aerosol production, *P*_*i*_, (or loss) is inherently not an equilibrium process, but many terms will reach a steady state in different situations. There are two relevant limits: one where condensation to suspended particles controls the vapour concentrations on a timescale given by the condensation sink , and one where losses, *k*_*i*_ (that is, wall losses), control those vapour concentrations. We are interested in the steady-state saturation ratios  and excess saturation ratio .

When losses control the steady-state, . If the suspended particles control the steady-state, the excess saturation ratio will be in steady state. A fraction of *P*_*i*_ will go to vapours and a fraction to the particles. The latter fraction will be approximately . is a key diagnostic for organic condensation. If , the condensation will be essentially ‘non-volatile’ ( will have no influence on the condensation), while if  then the condensation will be ‘semi-volatile’. Finally, if , species *i* cannot be an important driver of the condensation, as  cannot grow larger than *S*_*i*_ during net gas-phase production.

### Dynamic volatility-distribution modelling of aerosol growth

From ref. 15, where the yields were derived from the same experiments, we know the molar yield of HOMs to be roughly ~2.9% from α-pinene ozonolysis. The molar weight of the HOMs is on average twice the molar weight of α-pinene, and we approximate a mass yield of the HOMs of about 6%. The HOMs used include monomers, dimers and intermediate compounds as seen by the nitrate-CI-APi-TOF. The concentration of other neutral multimers was either too low or below detection limit (and thus also too low) to contribute significantly to the growth and were neglected in the model. The dynamic volatility-distribution model then condenses the observations into nine volatility bins ranging from C* = 10^−8^ μg m^−3^ to C* = 1 μg m^−3^. ELVOC and LVOC were defined as C* < 10^−4.5^ μg m^−3^ and 10^−4.5^ μg m^−3^ < C* < 10^−0.5^ μg m^−3^ respectively, which is slightly modified compared to ref. 18. This is justified as species with C* = 10^−4^ μg m^−3^(typically ELVOC) behaved rather like LVOC, that is, the condensation flux increases with diameter. In Fig. 1 we have seen that the measured HOMs alone cannot explain the observed growth in all size ranges. Therefore, a larger yield of C* = 1 μg m^−3^ was assumed (light shaded area in Extended Data Fig. 5a), which represents the compounds participating in the formation of the traditional secondary organic aerosol (SOA). Species with C* ≤ 10^−8^ μg m^−3^ were brought into one single bin with C* = 10^−8^ μg m^−3^. The CI-APi-TOF transmission calibration was multiplied by a factor of 1.3, which is within the transmission efficiency uncertainties. The resulting HOM distribution (in percentage) is displayed in Extended Data Fig. 5a.

Using this adjusted HOM distribution, we modelled the growth rate due to condensation assuming no Kelvin effect. Extended Data Fig. 6 shows that the model overestimates the early growth rate and substantially underestimates the observed particle growth rates at larger sizes (blue dashed line). In a next step we modified the charging efficiencies, to match the observation better. Our best result was achieved with values of [0.5, 0.4, 0.3, 0.1] for the VBS bins from 10^−4^ to 10^−1^ μg m^−3^, meaning that we increased the raw measured values by [2, 2.5, 3.3, and 10]. Still, it is not possible to describe the observations as depicted by the solid blue line in Extended Data Fig. 6.

Therefore it is essential to introduce the Kelvin effect to reproduce the observed growth rate. In the model we use a Kelvin diameter *D*_K_ = 3.75 nm. This corresponds to a surface tension of 23 mN m^−1^, which is a reasonable value for organics^64^. If we attempt to model the observed growth using the HOM volatility distribution in Extended Data Fig. 5a, Extended Data Fig. 6 shows that the model substantially underestimates the observed particle growth (pink dashed line), as expected.

The efficiency of HOM charging by the nitrate anion  depends upon the number and location of OOH groups^21^. As the probability of two OOH groups at optimal configuration is highest for the least volatile species (ELVOC), their charging efficiency is near unity. For products with higher volatility (LVOC) the efficiency decreases. Many of the oxidized monomers might still have a stiff carbon 4-ring backbone hindering an optimal cluster formation between two OOH groups and the nitrate ion. This decreased charging efficiency has yet to be experimentally quantified. Cycloalkene experiments indicate that the nitrate-CI-APi-TOF indeed underestimates the low-oxygenated compounds, if compared with the acetate-CIMS^65^, while the concentration for highly oxygenated compounds is similar. The ELVOC bins cannot be changed to a great extent as this would yield an overestimation in the growth rate at sizes below 3 nm.

Adjusting both the LVOC concentrations and the Kelvin term, it is possible to explain the observed size dependent behaviour in Fig. 3. Our best fit was achieved with charging efficiencies of [0.5, 0.25, 0.1, 0.1] for the VBS bins from 10^−4^ to 10^−1^ μg m^−3^ and a Kelvin diameter *D*_K_ = 3.75 nm. The final adjusted yields can be seen in Extended Data Fig. 5b, which displays the HOM fraction in the corresponding volatility bins (in percentage). Other tested Kelvin diameters (for example, *D*_K_ = 4.5 nm) yielded a slightly worse agreement with the measurements, the qualitative picture, however, remained the same. Increasing *D*_K_ requires an additional adjustment of the ELVOCs to match the observations, so that several parameter combinations will yield similar results. However, very large *D*_K_ are very unlikely, as there is not much space to increase the ELVOC concentration due to the nitrate-CI-APi-TOF measurement principle.

Here we do not attempt to constrain the volatility distribution exactly. We show that the distribution matters in the formation of particles. ELVOC condensation dominates the growth up to ~1.5 nm. Beyond this size, LVOC can contribute and drive the growth. It should be noted that the HOM distribution will change with chamber operating conditions (temperature, α-pinene concentration, particle concentration).

Here we only show two representative runs, but very different cases. We did not perform experiments with pre-existing particles in the chamber, at least not in such an amount to overcome the sink due to the wall (*k*_wall_ ≈ 10^−3^ s^−1^ versus *k*_cond_ ≈ 10^−4^ s^−1^ or lower). The wall loss does in some way simulate the sink due to pre-existing particles. The measured gas-phase concentration is a result of the existing sink and source terms. These terms will be somewhat different in the chamber compared to ambient conditions. Thus, we cannot say that under the same α-pinene and ozone concentrations the growth is the same. But, measuring the same volatility distribution of HOMs in the ambient (and at the same temperature) should yield similar results. The exact evolution of the particle size and the contribution of the volatility bins will always depend on the observed volatility distribution of the HOM species. The volatility distribution itself will depend on the temperature and the oxidants (for example, NO_*x*_ will hinder the formation of ELVOC, lowering the yield^17^). But the approach proposed here and the corresponding conclusion will still be applicable.

*Model details.* For the simulations we assumed a mono-disperse population of nucleated particles at an initial size of 1.2 nm mobility diameter or 0.9 nm physical diameter (which is approximately the monomer size). The key parameter is the concentration gradient (see equation (9)), which in turn reflects the differences in activity between the gas phase (the saturation ratio) and the particle phase (here the mass fraction). This can be seen in Extended Data Fig. 7a. The gas phase is characterized by the balance between the production rate of the α-pinene oxidation products and wall losses yielding a stable gas-phase saturation ratio. In contrast, the condensed phase activities drop as soon as the particles grow and the Kelvin effect decreases.

Looking at the excess saturation (Extended Data Fig. 7b), the least volatile species (mostly ELVOC) have a significant excess saturation at all times; the condensed phase activity is always much lower than the gas-phase saturation ratio. The more volatile species are near equilibrium at the beginning, only gradually (if ever) developing a significant driving force of condensation. The most volatile species are in equilibrium all of the time with a diminishing mass fraction in the condensed phase. For <2.5 nm, the particles are unstable, with the majority of their constituents showing activities . They can only grow as a consequence of the excess saturation ratio of the ELVOCs. If the production were rapidly stopped, the particles would evaporate. Extended Data Fig. 7b also shows the condensed phase mass fraction and thus the chemical composition of the particle. Particles <2.5 nm are mainly composed of ELVOC dominated by species with C* = 10^−8^ μg m^−3^. For larger particles the LVOC mass fraction increases until each contributes equally to the particle composition. The two most volatile bins never contribute substantially to the particle composition as their gas-phase saturation ratio is too low.

Extended Data Fig. 7c shows the absolute driving force of condensation and the equilibrium concentration of the different volatile species over the growing particles. Here, this transition from ELVOC to LVOC dominated growth is evident in the driving force of condensation. Owing to the stiff coupled differential equations tight tolerances on the solver are required for the solution to converge accurately.

### Appearance times and growth rate estimation of clusters and aerosols

The appearance times of clusters and aerosols allow us to investigate the growth process. Cluster and particle appearance times, defined as the 50% rise time of the concentration of a cluster or size channel^66^, were derived for APi-TOF, PSM, NAIS, DEG-CPCs, nRDMA and nano-SMPS. The corresponding diameters (leading edge diameter) were then plotted against the time. The temporal evolution is then representing the growth rate. For linear evolution, a linear fit was applied; the slope yields the growth rate. Extended Data Fig. 1 combines all the calculated appearance times for one example run. It shows an excellent agreement between the different methods and instruments.

To determine the appearance time for APi-TOF, NAIS, and PSM, concentrations in each size bin were analysed and the time when the concentration reaches 50% of its maximum value after the start of a nucleation experiment was determined and linked with the diameter midpoint of the size bin. The growth rate was obtained from a linear fit of the appearance times and the corresponding diameters. For the PSM the growth rate could be determined for the size range 1.5–3.2 nm. For the NAIS: (1) 1.4–3 nm, (2) 5–15 nm and (3) 15–30 nm. In the APi-TOF, appearance times of the monomers, dimers, trimers and tetramers were determined.

A normal (Gauss) function was applied to the size distribution data^2,67^. The position of the full-width at half-maximum (FWHM) was then defined as the 50% rise time. Nano-SMPS growth rates were determined for the following size ranges: (1) 5–15 nm, (2) 15–30 nm, (3) 30–60 nm and (4) >60 nm. In these size ranges, a constant growth rate for constant HOM concentration was observed, so we did not further differentiate these ranges in Fig. 1. For the nRDMA: (1) 1.1–3 nm, (2) 2–7 nm.

The DEG-CPC method was slightly different. In previous studies^6^, the 1% threshold of the CPC and the initial rise of the concentration was used to further extend the growth rate analysis to lower diameters. We decided to also use this approach for the DEG-CPCs. However, owing to the high noise, it was often difficult to determine the 1% rise time, thus the 5% rise time of the DEG-CPCs was used instead, yielding similar results.

*Growth rate uncertainties.* The method uncertainty is estimated^66^ to be approximately 50%. To consider the run-to-run uncertainty, we used *σ*_fit_, as retrieved from the linear fit uncertainty to determine the growth rate (GR). The overall uncertainty then scales as follows:The growth rates in Fig. 1c, d correlate reasonably well with the HOM concentration. Growth rates of larger sizes correlate with a Pearson’s correlation coefficient of 0.94, growth rates at smaller size with a Pearson’s correlation coefficient of 0.7. The lower correlation at the smaller sizes can be explained by the higher measurement uncertainty at these size ranges, compared to larger sizes.

### Parameterization of first steps of growth and global aerosol modelling

We are especially interested in the first steps of growth, that is, from the nucleated cluster size to 3 nm, as there the coagulation losses are highest. In the global model we use here^68^, nucleated clusters have a diameter of 1.7 nm, and particles must grow to 3 nm before being advected through the atmosphere in the nucleation mode. Therefore we parameterize the growth rate in the size range 1.7–3 nm. We use the size-resolved growth rates from the HOM volatility-distribution modelling results to derive a size-dependent parameterization. The Kelvin effect increases the growth rate with increasing size. The considered size range (1.7–3 nm) is small enough that we can approximate the dependence on the particle diameter *D*_p_ as linear. We thus parameterize the growth rate (in nm h^−1^) by fitting the two-dimensional function ([HOM] in cm^−3^, *D*_p_ in nm):to the HOM volatility-distribution modelling results, with the free parameters *k* = (5.2 ± 0.4) × 10^−11^ and *p* = 1.424 ± 0.004. Here the uncertainties are those from the fit only; they reflect how well the function describes the data but do not represent the full uncertainty in the parameterization. The parameterization is intended to describe the size-dependent growth that we observe, and does not necessarily reflect the underlying mechanism. Therefore, extrapolations to very high values (>5 × 10^8^ cm^−3^) and low values (<2 × 10^6^ cm^−3^) may not be reliable, as it is likely that the parameterized growth rates deviate from the true growth rates. Such high biogenic HOM values, however, are not expected in the field and should not impact the global modelling results. Conversely, low HOM concentrations far below 2 × 10^6^ cm^−3^ are expected far from sources of terpenes, especially over oceans and the upper free troposphere. From Fig. 1 it is evident that the growth rate at [HOM] <2 × 10^6^ cm^−3^ is <1 nm h^−1^. Under these conditions, growth is driven by condensation of sulfuric acid, and uncertainties in the parameterization of the very small organic contribution are not expected to affect the results significantly.

This parameterization provides a refined estimate of the growth rate between 1.7 and 3 nm, which is appropriate for models of atmospheric aerosol that treat SOA condensation kinetically. To implement the parameterization, a mechanism and yield for the production of HOMs is required. In our model, HOMs are simulated as being produced directly from the oxidation of monoterpenes (MT) and lost to the condensation sink (CS) in a steady-state approximation:where *Y*_1_, the yield of HOMs from the ozonolysis of monoterpenes, is 2.9%, and *Y*_2_, the yield from the OH-oxidation, is 1.2%. The yields were determined from the nitrate-CI-APi-TOF and PTR-TOF measurements in the CLOUD chamber^15^. The constants *k*_1_ and *k*_2_ are the temperature dependent reaction rate constants of α-pinene with ozone and hydroxyl radicals, respectively^69^. Thus the numerator of equation (16) represents the production of HOMs and the denominator the losses.

We do not quote a similar parameterization for growth rates at larger sizes, because it is clear that the nitrate-CI-APi-TOF does not see all of the more volatile molecules that condense onto larger particles, many more compounds are likely to participate than those present in the CLOUD chamber, and at these larger sizes the kinetic condensation approach should be complemented by an equilibrium partitioning treatment (for example, ref. 70).

This parameterization represents pure organic growth resulting from biogenic emissions. In the ambient atmosphere, additional organic and inorganic precursors such as sulfuric acid, ammonia, amines and anthropogenic VOCs are also present and influence the growth rate, in addition to the different oxidants. Also temperature and relative humidity could influence the observed growth rates. So, while this parameterization represents a significant advance on the current state of the art, it should not be considered complete. Furthermore, we only consider the size range 1.7 to 3 nm, as the growth in this size range is most decisive for the fate of the freshly nucleated particle^4^.

The parameterization of initial particle growth is incorporated in the global aerosol model GLOMAP-mode^68^, an extension to the TOMCAT chemical transport model^71^. GLOMAP includes representations of particle formation, growth via coagulation, condensation and cloud processing, wet and dry deposition and in/below cloud scavenging. The horizontal resolution is 2.8 × 2.8 degrees and there are 31 vertical sigma-pressure levels extending from ground level to 10 hPa. Aerosol in the model is formed of four components: black carbon, organic carbon, sea salt and sulfate, and is advected through the atmosphere in seven log-normal size modes. These are hygroscopic nucleation, Aitken, accumulation and coarse modes, and non-hygroscopic Aitken, accumulation and coarse modes. Formation of secondary particles in the model is based on CLOUD measurements of ternary H_2_SO_4_-organic-H_2_O nucleation detailed in ref. 25 and on a parameterization of binary H_2_SO_4_-H_2_O nucleation^72^. Simulations are run for the year 2008.

In the aerosol model, particles grow by irreversible condensation of monoterpene oxidation products and sulfuric acid. Monoterpene emissions in the model are taken from the database of ref. 73. Our measurements^15^ provide HOM yields of 2.9% from the oxidation of α-pinene by ozone and 1.2% from the hydroxyl radical. In ref. 58 a substantially higher HOM yield was observed from endocyclic monoterpenes such as α-pinene than from exocylic monoterpenes. These two types are roughly equally abundant in the atmosphere. Thus, we account for this by dividing our measured yields by two. In the light of these results, we also divide the organic nucleation rate of ref. 25 by two, since it also assumed all terpenes were represented by α-pinene in the atmosphere. Above 3 nm in diameter, a fixed 13% of the oxidation products of monoterpenes with OH, O_3_ and NO_3_ (assuming the reaction rates of α-pinene) condense irreversibly onto aerosol particles at the kinetic limit. These oxidized organic molecules are referred to as SORG and are advected through the troposphere as a tracer in the model, while the HOM concentration is calculated assuming a steady state as described earlier. Below 3 nm, organic molecules condense onto particles according to the parameterization, while sulfuric acid molecules condense at the kinetic limit (collision-limited), which is approximately:Additional model runs were performed with no organics participating in the initial growth, and with non-volatile size-dependent growth of particles between 1.7 and 3 nm due to condensation of SORG multiplied by the factor determined in ref. 30 for the parameterization of ref. 3,where *D*_p_ is the particle diameter in nm and the correction is only applied to particles below 2.5 nm. We note that the SORG in GLOMAP is produced with a 13% yield while that in GEOS-chem is produced with a 10% yield. The growth rates in these three cases are shown in Extended Data Fig. 9, together with the HOM concentration in the model.

## Figures and Tables

**Extended Data Table 1:**
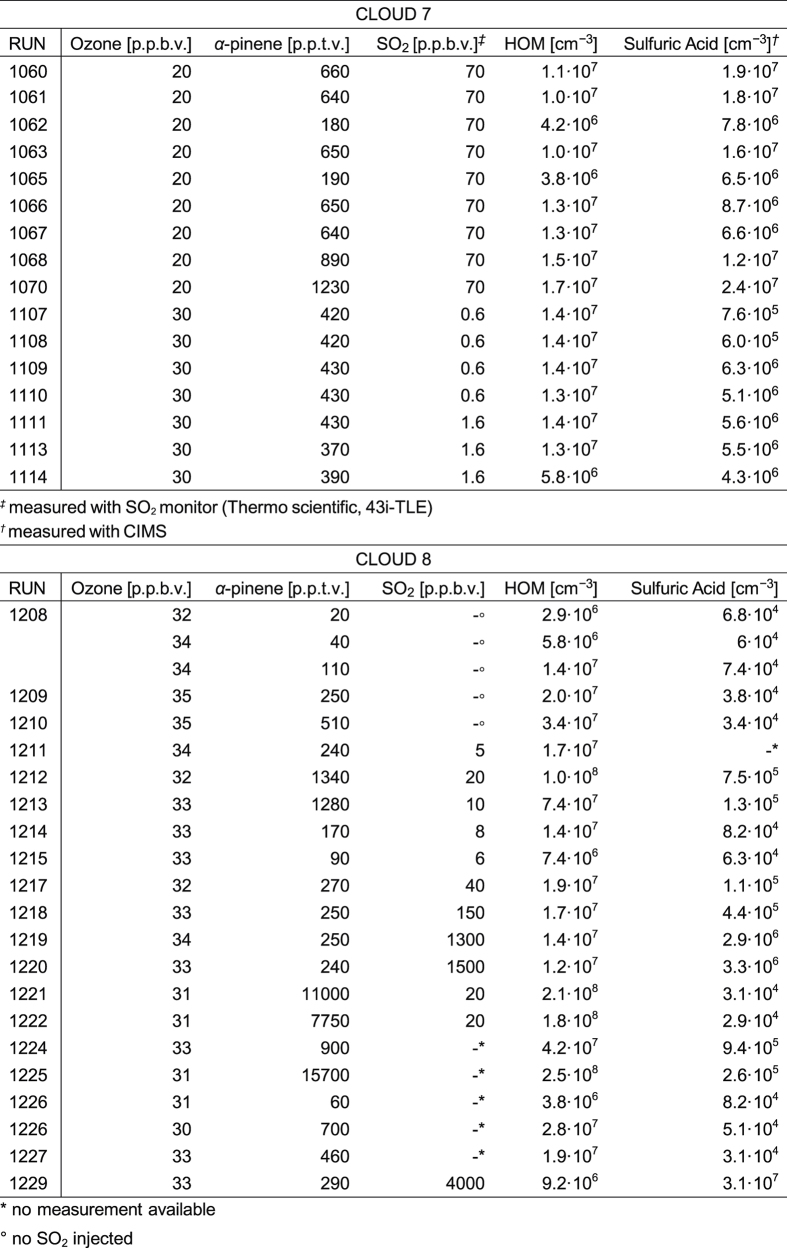
Summary of CLOUD runs during CLOUD7 and CLOUD8

Each run consisted of several stages (increasing gases, steady-sate, changing charging state of chamber, see also Extended Data Fig. 8), here only the steady-state plateau values are indicated.

## Related audio

Davide Castelvecchi talks to the researchers making clouds in the lab.

## PowerPoint slides

PowerPoint slide for Fig. 1
PowerPoint slide for Fig. 2
PowerPoint slide for Fig. 3
PowerPoint slide for Fig. 4

## Source data

Source data to Fig. 1
Source data to Fig. 2
Source data to Fig. 3

## References

[1] Merikanto J, Spracklen DV, Mann GW, Pickering SJ, Carslaw KS (2009). Impact of nucleation on global CCN. Atmos. Chem. Phys..

[2] Kulmala M (2013). Direct observations of atmospheric aerosol nucleation. Science.

[3] Kuang C (2012). Size and time-resolved growth rate measurements of 1 to 5 nm freshly formed atmospheric nuclei. Atmos. Chem. Phys..

[4] Lehtinen KE, Dal Maso M, Kulmala M, Kerminen V-M (2007). Estimating nucleation rates from apparent particle formation rates and vice versa: revised formulation of the Kerminen-Kulmala equation. J. Aerosol Sci..

[5] Nieminen T, Lehtinen KEJ, Kulmala M (2010). Sub-10 nm particle growth by vapor condensation-effects of vapor molecule size and particle thermal speed. Atmos. Chem. Phys..

[6] Riccobono F (2012). Contribution of sulfuric acid and oxidized organic compounds to particle formation and growth. Atmos. Chem. Phys..

[7] Riipinen I (2012). The contribution of organics to atmospheric nanoparticle growth. Nat. Geosci..

[8] Smith JN (2008). Chemical composition of atmospheric nanoparticles formed from nucleation in Tecamac, Mexico: evidence for an important role for organic species in nanoparticle growth. Geophys. Res. Lett..

[9] Laaksonen A (2008). The role of VOC oxidation products in continental new particle formation. Atmos. Chem. Phys..

[10] Donahue NM (2013). How do organic vapors contribute to new-particle formation?. Faraday Discuss..

[11] Zhao J, Ortega J, Chen M, McMurry P, Smith J (2013). Dependence of particle nucleation and growth on high-molecular-weight gas-phase products during ozonolysis of *α*-pinene. Atmos. Chem. Phys..

[12] Donahue NM, Trump ER, Pierce JR, Riipinen I (2011). Theoretical constraints on pure vapor-pressure driven condensation of organics to ultrafine particles. Geophys. Res. Lett..

[13] Pierce JR (2011). Quantification of the volatility of secondary organic compounds in ultrafine particles during nucleation events. Atmos. Chem. Phys..

[14] Kulmala M, Kerminen V-M, Anttila T, Laaksonen A, O’Dowd CD (2004). Organic aerosol formation via sulphate cluster activation. J. Geophys. Res. D.

[15] Kirkby, J. et al. Ion-induced nucleation of pure biogenic particles. *Nature* **533**, 10.1038/nature17953 (2016) 10.1038/nature17953 PMC8384037 27225125

[16] Jokinen T (2015). Production of extremely low volatile organic compounds from biogenic emissions: measured yields and atmospheric implications. Proc. Natl Acad. Sci. USA.

[17] Ehn M (2014). A large source of low-volatility secondary organic aerosol. Nature.

[18] Donahue NM, Kroll JH, Pandis SN, Robinson AL (2012). A two-dimensional volatility basis set — part 2: diagnostics of organic-aerosol evolution. Atmos. Chem. Phys..

[19] Lovejoy ER, Curtius J, Froyd KD (2004). Atmospheric ion-induced nucleation of sulfuric acid and water. J. Geophys. Res. D.

[20] Pankow JF, Asher WE (2008). SIMPOL. 1: A simple group contribution method for predicting vapor pressures and enthalpies of vaporization of multifunctional organic compounds. Atmos. Chem. Phys..

[21] Hyttinen N (2015). Modeling the charging of highly oxidized cyclohexene ozonolysis products using nitrate-based chemical ionization. J. Phys. Chem. A.

[22] Presto AA, Donahue NM (2006). Investigation of *α*-pinene + ozone secondary organic aerosol formation at low total aerosol mass. Environ. Sci. Technol..

[23] Trump ER, Donahue NM (2014). Oligomer formation within secondary organic aerosols: equilibrium and dynamic considerations. Atmos. Chem. Phys..

[24] Schobesberger S (2013). Molecular understanding of atmospheric particle formation from sulfuric acid and large oxidized organic molecules. Proc. Natl Acad. Sci. USA.

[25] Riccobono F (2014). Oxidation products of biogenic emissions contribute to nucleation of atmospheric particles. Science.

[26] Wang L (2010). Atmospheric nanoparticles formed from heterogeneous reactions of organics. Nat. Geosci..

[27] Yli-Juuti T (2011). Growth rates of nucleation mode particles in Hyytiälä during 2003–2009: variation with particle size, season, data analysis method and ambient conditions. Atmos. Chem. Phys..

[28] Häkkinen SAK (2013). Semi-empirical parameterization of size-dependent atmospheric nanoparticle growth in continental environments. Atmos. Chem. Phys..

[29] Bianchi, F. et al. New particle formation in the free troposphere: a question of chemistry and timing. *Science* **352**, 10.1126/science.aad5456 (2016) 10.1126/science.aad5456 27226488

[30] D’Andrea SD (2013). Understanding global secondary organic aerosol amount and size-resolved condensational behavior. Atmos. Chem. Phys..

[31] Kirkby J (2011). Role of sulphuric acid, ammonia and galactic cosmic rays in atmospheric aerosol nucleation. Nature.

[32] Duplissy J (2016). Effect of ions on sulfuric acid-water binary particle formation: 2. Experimental data and comparison with qc-normalized classical nucleation theory. J. Geophys. Res. Atmos..

[33] Kupc A (2011). A fibre-optic UV system for H_2_SO_4_ production in aerosol chambers causing minimal thermal effects. J. Aerosol Sci..

[34] Duplissy J (2010). Results from the CERN pilot CLOUD experiment. Atmos. Chem. Phys..

[35] Voigtländer J, Duplissy J, Rondo L, Kürten A, Stratmann F (2012). Numerical simulations of mixing conditions and aerosol dynamics in the CERN CLOUD chamber. Atmos. Chem. Phys..

[36] Schnitzhofer R (2014). Characterisation of organic contaminants in the CLOUD chamber at CERN. Atmos. Meas. Tech..

[37] Bianchi F, Dommen J, Mathot S, Baltensperger U (2012). On-line determination of ammonia at low pptv mixing ratios in the CLOUD chamber. Atmos. Meas. Tech..

[38] Jokinen T (2012). Atmospheric sulphuric acid and neutral cluster measurements using CI-APi-TOF. Atmos. Chem. Phys..

[39] Graus M, Müller M, Hansel A (2010). High resolution PTR-TOF: quantification and formula confirmation of VOC in real time. J. Am. Soc. Mass Spectrom..

[40] Junninen H (2010). A high-resolution mass spectrometer to measure atmospheric ion composition. Atmos. Meas. Tech..

[41] Kürten A, Rondo L, Ehrhart S, Curtius J (2012). Calibration of a chemical ionization mass spectrometer for the measurement of gaseous sulfuric acid. J. Phys. Chem. A.

[42] Cheng, Y.-S. in * Aerosol Measurement: Principles, Techniques, and Applications * (eds Kulkarni, P. et al.) 569–601 (John Wiley & Sons, 2001)

[43] Heinritzi M (2016). Characterization of the mass-dependent transmission efficiency of a CIMS. Atmos. Meas. Tech..

[44] Möhler O, Reiner TH, Arnold F (1992). The formation of  by gas phase ion-molecule reactions. J. Chem. Phys..

[45] Kürten A, Rondo L, Ehrhart S, Curtius J (2011). Performance of a corona ion source for measurement of sulfuric acid by chemical ionization mass spectrometry. Atmos. Meas. Tech..

[46] Brunelli NA, Flagan RC, Giapis KP (2009). Radial differential mobility analyzer for one nanometer particle classification. Aerosol Sci. Technol..

[47] Wang J, McNeill VF, Collins DR, Flagan RC (2002). Fast mixing condensation nucleus counter: application to rapid scanning differential mobility analyzer measurements. Aerosol Sci. Technol..

[48] Jiang J (2011). Transfer functions and penetrations of five differential mobility analyzers for sub-2 nm particle classification. Aerosol Sci. Technol..

[49] Wang SC, Flagan RC (1990). Scanning electrical mobility spectrometer. Aerosol Sci. Technol..

[50] Kulkarni, P., Baron, P. A. & Willeke, K. *Aerosol Measurement: Principles, Techniques, and Applications* (John Wiley & Sons, 2011)

[51] Mirme S, Mirme A (2013). The mathematical principles and design of the NAIS–a spectrometer for the measurement of cluster ion and nanometer aerosol size distributions. Atmos. Meas. Tech..

[52] Asmi E (2009). Results of the first air ion spectrometer calibration and intercomparison workshop. Atmos. Chem. Phys..

[53] Gagné S (2011). Intercomparison of air ion spectrometers: an evaluation of results in varying conditions. Atmos Meas. Tech..

[54] Wimmer D (2013). Performance of diethylene glycol-based particle counters in the sub-3 nm size range. Atmos. Meas. Tech..

[55] Iida K, Stolzenburg MR, McMurry PH (2009). Effect of working fluid on sub-2 nm particle detection with a laminar flow ultrafine condensation particle counter. Aerosol Sci. Technol..

[56] Vanhanen J (2011). Particle size magnifier for nano-CN detection. Aerosol Sci. Technol..

[57] Rissanen MP (2014). The formation of highly oxygenated multifunctional products in the ozonolysis of cyclohexene. J. Am. Chem. Soc..

[58] Jokinen T (2014). Rapid autoxidation forms highly oxidized RO_2_ radicals in the atmosphere. Angew. Chem. Int. Ed..

[59] Mentel T (2015). Formation of highly oxidized multifunctional compounds: autoxidation of peroxy radicals formed in the ozonolysis of alkenes deduced from structure product relationships. Atmos. Chem. Phys..

[60] Zhang D, Zhang R (2005). Ozonolysis of *α*-pinene and *β*-pinene: kinetics and mechanism. J. Chem. Phys..

[61] Seinfeld, J. H. & Pandis, S. N. *Atmospheric Chemistry and Physics: from Air Pollution to Climate Change* (John Wiley & Sons, 2006)

[62] Fuchs, N. A. & Sutugin, A. G. *Coagulation rate of Highly Dispersed Aerosols* (Ann Arbor Science, 1970)

[63] Pankow JF (1994). An absorption model of gas/particle partitioning of organic compounds in the atmosphere. Atmos. Environ..

[64] Korosi G, Kovats ES (1981). Density and surface tension of 83 organic liquids. J. Chem. Eng. Data.

[65] Berndt T (2015). Gas-phase ozonolysis of cycloalkenes: formation of highly oxidized RO_2_ radicals and their reactions with NO, NO_2_, SO_2_, and other RO_2_ radicals. J. Phys. Chem. A.

[66] Lehtipalo K (2014). Methods for determining particle size distribution and growth rates between 1 and 3 nm using the particle size magnifier. Boreal Environ. Res..

[67] Kulmala M (2004). Initial steps of aerosol growth. Atmos. Chem. Phys..

[68] Mann GW (2010). Description and evaluation of GLOMAP-mode: a modal global aerosol microphysics model for the UKCA composition-climate model. Geoscientific Model Dev..

[69] McNaught, A. D. & Wilkinson, A. *Compendium Of Chemical Terminology* Vol. 1669 (Blackwell Science, 1997)

[70] Riipinen I (2011). Organic condensation: a vital link connecting aerosol formation to cloud condensation nuclei (CCN) concentrations. Atmos. Chem. Phys..

[71] Chipperfield MP (2006). New version of the TOMCAT/SLIMCAT off-line chemical transport model: Intercomparison of stratospheric tracer experiments. Q. J. R. Meteorol. Soc..

[72] Kulmala M, Laaksonen A, Pirjola L (1998). Parameterizations for sulfuric acid/water nucleation rates. J. Geophys. Res. D.

[73] Guenther A (1995). A global model of natural volatile organic compound emissions. J. Geophys. Res. D.

[74] Kurtén T (2015). Computational study of hydrogen shifts and ring-opening mechanisms in *α*-pinene ozonolysis products. J. Phys. Chem. A.

