# Supplementary material for: The role of low-volatility organic compounds in initial particle growth in the atmosphere
Source: Nature. 2016 May 25;533(7604):527–31. doi: 10.1038/nature18271 (PMC8384036; doi:10.1038/nature18271)
Supplement: Supplementary file 2 — PowerPoint slide for Fig. 1 [file 41586_2016_BFnature18271_MOESM135_ESM.ppt]

## Slide 1
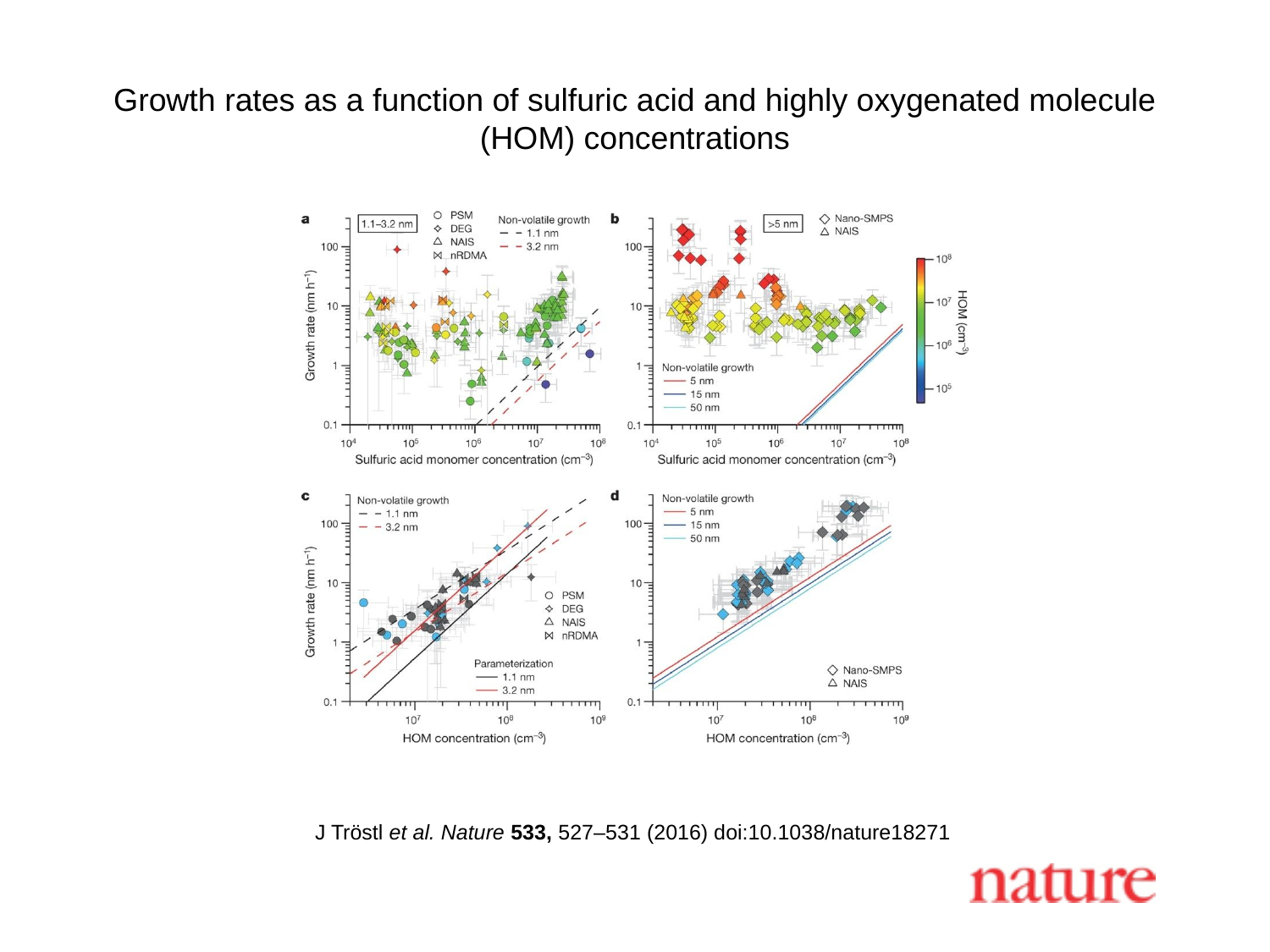

# Growth rates as a function of sulfuric acid and highly oxygenated molecule (HOM) concentrations
J Tröstl et al. Nature 533, 527–531 (2016) doi:10.1038/nature18271
