# Supplementary material for: The role of low-volatility organic compounds in initial particle growth in the atmosphere
Source: Nature. 2016 May 25;533(7604):527–31. doi: 10.1038/nature18271 (PMC8384036; doi:10.1038/nature18271)
Supplement: Supplementary file 5 — PowerPoint slide for Fig. 4 [file 41586_2016_BFnature18271_MOESM141_ESM.ppt]

## Slide 1
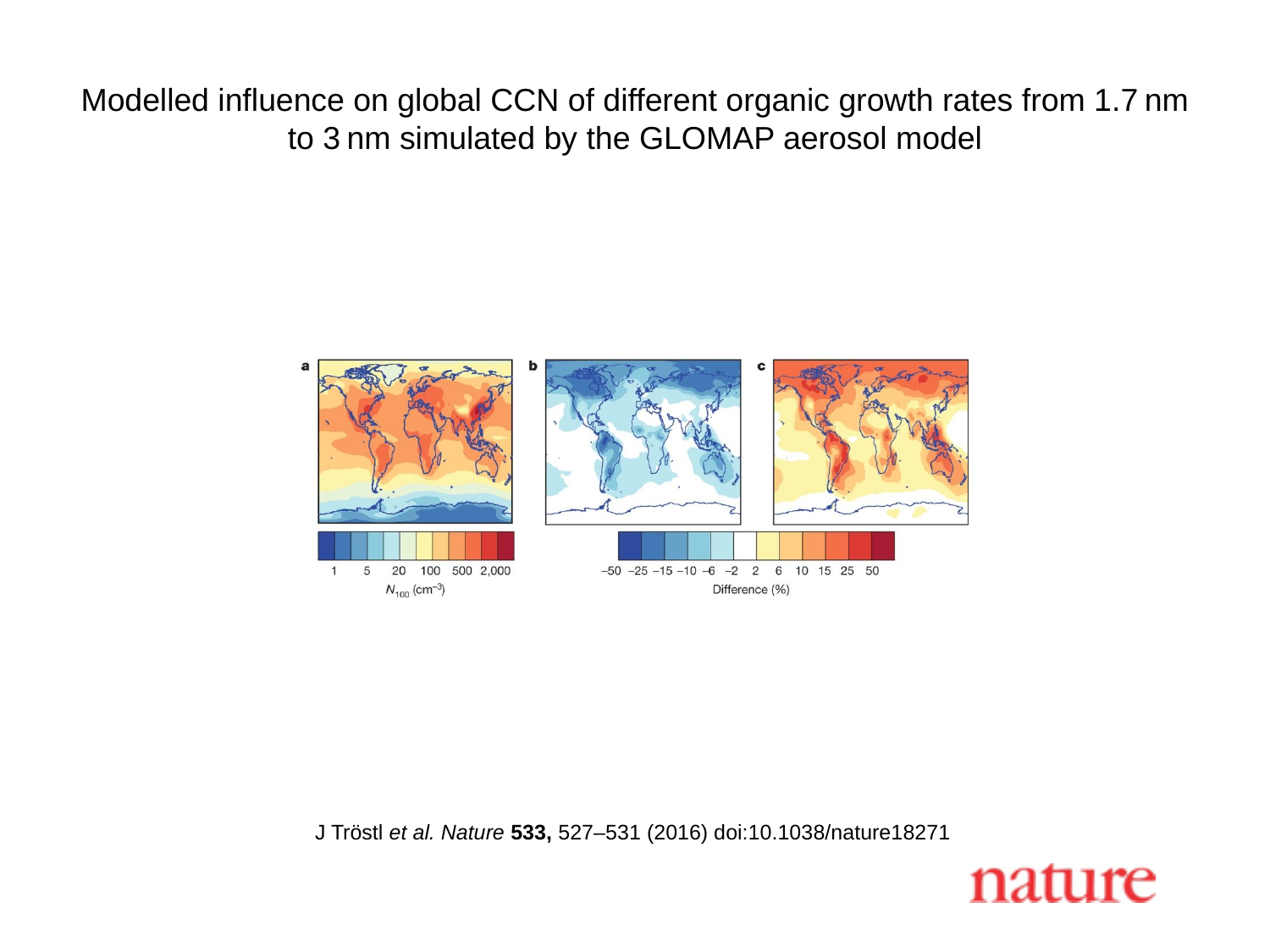

# Modelled influence on global CCN of different organic growth rates from 1.7 nm to 3 nm simulated by the GLOMAP aerosol model
J Tröstl et al. Nature 533, 527–531 (2016) doi:10.1038/nature18271
